# Supplementary figures and images for: Human nucleolar protein SURF6/RRP14 participates in early steps of pre-rRNA processing
Source: PLoS One. 2023 Jul 14;18(7):e0285833. doi: 10.1371/journal.pone.0285833 (PMC10348582; doi:10.1371/journal.pone.0285833)

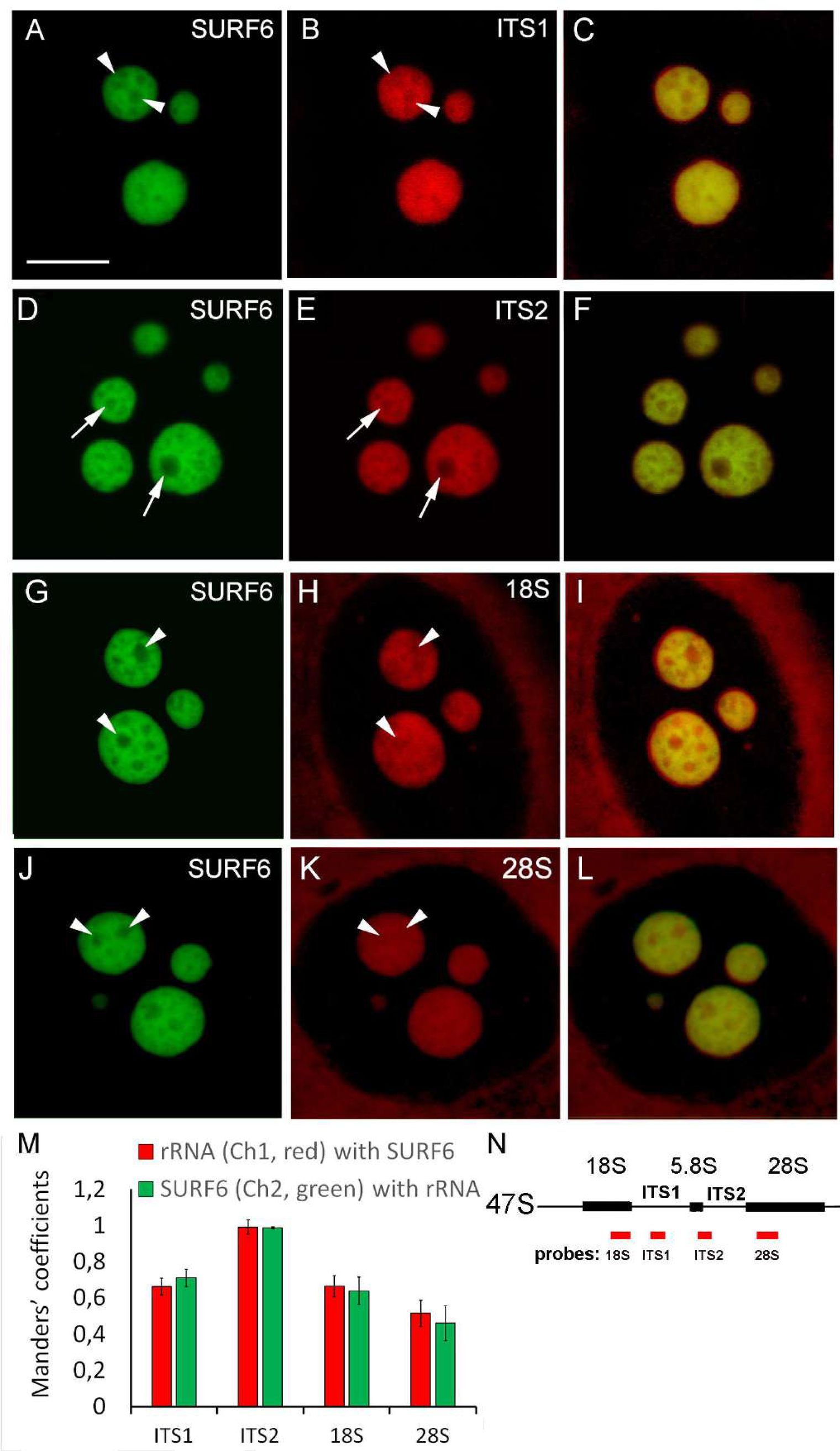

Supplement: S1 Fig — The confocal images of the nuclei stained with anti-SURF6 antibody (A, D, G, J; green) or FISH hybridized with the probes specific to ITS1 (B), ITS2 (E), 18S rRNA (H), 28S rRNA (K) (red). Panels (C, F, I, L)–merged images of SURF6 and FISH confocal sections. Arrows point to the areas of nucleoli where the difference between FISH and immunostaining are clearly visible. Representative images are shown. (M) Quantitation of overlap (in %) between SURF6 and FISH presented as bar graph. (N) Structure of human 47S pre-rRNA, red lines correspond to positions of the FISH probes. (TIF) [file pone.0285833.s001.tif]
