# Supplementary material for: Human nucleolar protein SURF6/RRP14 participates in early steps of pre-rRNA processing
Source: PLoS One. 2023 Jul 14;18(7):e0285833. doi: 10.1371/journal.pone.0285833 (PMC10348582; doi:10.1371/journal.pone.0285833)
Supplement: S1 Data — (DOCX) [file pone.0285833.s002.docx]

**Table 1**

**Suplimentary_ Figure 1**  (rRNA and SURF6 co-localization)

Co-localization rRNA (Ch1, red) with SURF6, %

|  | Mean | SEM |
| --- | --- | --- |
| ITS1 | 66,3% | 4,6% |
| ITS2 | 99,1% | 0,4% |
| 18S rRNA | 66,4% | 5,9% |
| 28S rRNA | 51,4% | 7,2% |

Co-localization SURF6 (Ch2, green) with rRNA, %

|  | |  |
| --- | --- | --- |
|  | Mean | SEM |
| ITS1 | 71,1% | 4,8% |
| ITS2 | 98,8% | 0,5% |
| 18S рРНК | 63,9% | 7,5% |
| 28S рРНК | 46,1% | 9,6% |

**Table 2**

**Figure 1-2 A, B** (western blots analysis)

| 1 | SEM | Control |  |
| --- | --- | --- | --- |
| 4,5 | 0,23 | Overexpression | |
|  |  |  |  |
|  |  |  |  |
| 1 |  | Control |  |
| 0,13 | 0,05 | siRNA |  |

**Table 3**

**Figure 4 A, B,D,E,G** (northern blots analysis)

| **overexpression** | | | | | |
| --- | --- | --- | --- | --- | --- |
| ***ITS1*** | | | | | |
| № Raw_fig | Raw_fig3 | Raw_fig6 | Raw_fig8 | **Mean** | **SEM** |
| 47/45S | 0,98 | 1,02 | 1,08 | 1,03 | 0,02 |
| 41S | 1,14 | 2,72 | 3,33 | 2,4 | 0,66 |
| 30S | 0,58 | 0,58 | 0,86 | 0,67 | 0,09 |
| 26S | 0,59 | 0,82 | 0,77 | 0,73 | 0,07 |
| 21S | 0,64 | 0,83 | - | 0,73 | 0,09 |
| 18SE | 0,92 | 1,03 | 0,66 | 0,87 | 0,11 |
| ***ITS2*** | | | | | |
| № Raw_fig | Raw_fig3 | Raw_fig6 | Raw_fig8 | **Mean** | **SEM** |
| 47/45S | 2,31 | 0,86 | 1,03 | 1,4 | 0,47 |
| 41S | 3,05 | 0,89 | 0,89 | 1,61 | 0,73 |
| 32S | 2,51 | 1,15 | 0,78 | 1,48 | 0,54 |
| 12S | 1,34 | 1,37 | 0,99 | 1,23 | 0,13 |
|  |  |  |  |  |  |
| **Knockdown** | | | | | |
| ***ITS1*** | | | | | |
| № Raw_fig | Raw_fig.4 | Raw-fig. 5 | Raw-fig7 | **Mean** | **SEM** |
| 47/45S | 1,04 | 1,08 | 1,07 | **1,05** | 0,01 |
| 41S | 1,49 | 1,50 | 1,50 | **1,49** | 0,007 |
| 30S | 0,35 | 0,64 | 0,49 | **0,49** | 0,01 |
| 26S | 0,53 | 0,53 | 0,38 | **0,47** | 0,01 |
| 21S | 0,51 | 0,85 | 0,61 | **0,65** | 0,01 |
| 18SE | 1,76 | 1,54 | 2,07 | **1,78** | 0,15 |
| ***ITS2*** | | | | | |
| № Raw_fig | Raw_fig4 | Raw-fig5 | Raw-fig7 | **Mean** | **SEM** |
| 47/45S | 0,99 | 1,05 | 0,48 | **0,84** | 0,18 |
| 41S | 1,22 | 1,14 | 1,49 | **1,28** | 0,11 |
| 32S | 0,65 | 0,91 | 1,18 | **0,91** | 0,15 |
| 12S | 0,99 | 0,74 | 0,64 | **0,78** | 0,10 |

**Table 4**

**Figure 4 C, F,H** (northern blots analysis)

| **P53-** | | | | | | |
| --- | --- | --- | --- | --- | --- | --- |
| ***ITS1*** | | | | | | |
| № Raw_fig | Raw_fig11 | Raw-fig9 | Raw-fig10 |  | **Mean** | **SEM** |
| 47/45S | 1,11 | 0,99 | 1,10 | 1,24 | **1,12** | 0,05 |
| 41S | 1,81 | 1.28 | 1.18 | 0.96 | **1.31** | 0.18 |
| 30S | 0.88 | 0.95 | 1.24 | 0.97 | **1,01** | 0,08 |
| 26S | 0.79 | 0.69 | 0.97 | 1.07 | **0,88** | 0,08 |
| 21S | 0.68 | 0.92 | 0.79 | 1.04 | **0,86** | 0,08 |
| 18SE |  | 0.84 | 3.6 | 0.83 | **1,76** | 0,34 |
| ***ITS2*** | | | | | | |
| № Raw_fig | Raw_fig11 | Raw-fig10 | Raw-fig9 |  | **Mean** | **SEM** |
| 47/45S | 0.99 | 1.41 | 0.98 |  | **1.13** | 0.12 |
| 41S | 1.4 | 1.18 | 1.06 |  | **1.21** | 0.08 |
| 32S | 1.22 | 0.93 | 0.96 |  | **1,04** | 0,08 |
| 12S | 1.16 | 0.89 | 0.93 |  | **0,99** | 0,07 |
| **P53+** | | | | | | |
| ***ITS1*** | | | | | | |
| № Raw_fig | Raw_fig11 | Raw-fig9 | Raw-fig10 | № Raw_fig | **Mean** | **SEM** |
| 47/45S | 0,40 | 1,01 | 0,84 | 0,83 | **0,77** | 0,13 |
| 41S | 0,56 | 1,01 | 0,76 | 0,81 | **0,79** | 0,09 |
| 30S | 0,54 | 0,98 | 1,07 | 0,94 | **0,88** | 0,12 |
| 26S | 0,7 | 0,72 | 0,93 | 0,80 | **0,79** | 0,05 |
| 21S | 0,75 | 0,68 | 0,58 | 1,04 | **0,76** | 0,09 |
| 18SE |  | 0,69 | 0,33 | 0,82 | **0,46** | 0,18 |
| ***ITS2*** | | | | | | |
| № Raw_fig | Raw_fig11 | Raw-fig9 | Raw-fig10 |  | **Mean** | **SEM** |
| 47/45S | 0,59 | 1,13 | 0,89 |  | **0,87** | 0,13 |
| 41S | 0,62 | 0,71 | 0,76 |  | **0,7** | 0,03 |
| 32S | 0,65 | 0,68 | 0,85 |  | **0,73** | 0,05 |
| 12S | 0,63 | 0,79 | 0,68 |  | **0,7** | 0,04 |

**Table 5**

**Figure 4 J** (RAMP analysis)

|  | knockdown | | overexpression | |
| --- | --- | --- | --- | --- |
|  | Mean | SEM | Mean | SEM |
| 12S/32S | **-0,21** | -0,04 | **-0,27** | -0,09 |
| 32S/41S | **-0,64** | -0,11 | **-0,23** | -0,10 |
| 32S/47S | **-0,29** | -0,05 | **0,29** | 0,12 |
| 18SE/41S | **0,36** | 0,04 | **-1** | -0,29 |
| 18SE/21S | **1,46** | 0,26 | **0,25** | 0,047 |
| 21S/26S | **0,44** | 0,08 | **0** | 0 |
| 26S/30S | **-0,03** | -0,01 | **0,12** | 0,04 |
| 30S/45S | **-1,17** | -0,20 | **-0,85** | -0,20 |
| 41S/47S | **0,35** | 0,02 | **0,52** | 0,17 |
| 12S/32S | **-0,21** | -0,04 | **-0,27** | -0,09 |

**Table 6**

**Figure 4 K** (RAMP analysis)

|  | P53- | | P53+ | |
| --- | --- | --- | --- | --- |
|  | Mean | SEM | Mean | SEM |
| 12S/32S | **-0,07** | -0,003 | **-0,06** | -0,02 |
| 32S/41S | **-0,33** | -0,07 | **-0,11** | -0,06 |
| 32S/47S | **-0,10** | 0,04 | **-0,08** | -0,13 |
| 41S/47'S | **0,10** | 0,02 | **-0,32** | 0,17 |
| 18SE/21S | **1,04** | 0,34 | **-0,72** | 0,24 |
| 21S/26S | **-0,04** | -0,01 | **-0,05** | 0,09 |
| 26S/30S | **-0,19** | 0,02 | **-0,16** | -0,09 |
| 18SE/41S | **0,42** | 0,12 | **-0,78** | 0,24 |
| 30S/45S | **-0,15** | 0,04 | **0,20** | -0,06 |
| 41S/47S | **0,23** | 0,16 | **0,03** | -0,07 |

**Table 7**

**Figure 5 A, B,C,D** (HeLa cell cycle under knockdown and overexpression of SURF6)

Knockdown conditions

|  | G1/G0 | | S | | G2/M | |
| --- | --- | --- | --- | --- | --- | --- |
| 24h | Control | knockdown | Control | knockdown | Control | knockdown |
|  | 73,1 | 66,6 | 19,2 | 25,6 | 7,7 | 7,7 |
|  | 78,5 | 70,8 | 15,9 | 22 | 5,6 | 7,1 |
|  | 79,5 | 71 | 14,7 | 22,2 | 5,8 | 6,8 |
|  | 57,8 | 55,2 | 34,7 | 30,1 | 10,2 | 12,1 |
| **Mean** | **72,225** | **65,9** | **21,125** | **24,975** | **7,325** | **8,425** |
| SEM | 1,721434 | 1,24231 | 1,165118 | 1,011599 | 0,579511 | 0,229129 |

|  | G1/G0 | | S | | G2/M | |
| --- | --- | --- | --- | --- | --- | --- |
| 48h | Control | knockdown | Control | knockdown | Control | knockdown |
|  | 81,6 | 77,1 | 12,5 | 17 | 5,9 | 5,3 |
|  | 83,8 | 79,5 | 11,4 | 16,6 | 4,9 | 3,9 |
|  | 82,5 | 78,2 | 10,1 | 16,9 | 7,4 | 4,9 |
|  | 75,2 | 64,7 | 21,2 | 29,2 | 3,6 | 6,1 |
| **Mean** | **80,775** | **74,875** | **13,8** | **19,925** | **5,45** | **5,05** |
| SEM | 0,553022 | 0,600694 | 0,600694 | 0,104083 | 0,629153 | 0,360555 |

|  | G1/G0 | | S | | G2/M | |
| --- | --- | --- | --- | --- | --- | --- |
| 72h | Control | knockdown | Control | knockdown | Control | knockdown |
|  | 85,3 | 72,7 | 10,4 | 22,5 | 4,3 | 4,7 |
|  | 82,5 | 75,3 | 14,2 | 24 | 3,3 | 0,7 |
|  | 80,4 | 64,7 | 14,4 | 31,4 | 5,2 | 3,6 |
| **Mean** | **82,73333** | **70,9** | **13** | **25,96667** | **4,266667** | **3** |
| SEM | 1,419311 | 3,189566 | 1,301281 | 2,750959 | 0,548736 | 1,193035 |

**Table 8**

**Figure 5 E,F,G** (HeLa cell cycle under knockdown and overexpression of SURF6)

Overexpression conditions

|  | HeLa |  |  |  |  |  |
| --- | --- | --- | --- | --- | --- | --- |
|  | G1/G0 | | S | | G2/M | |
|  | Control | overexpression | Control | overexpression | Control | overexpression |
|  | 45,3 | 44,1 | 44,5 | 42,1 | 10,3 | 13,8 |
|  | 45,5 | 48,5 | 47,7 | 42,2 | 6,8 | 9,3 |
|  | 57,9 | 59,7 | 33,6 | 33,2 | 8,5 | 7,2 |
| **Mean** | **49,56667** | **50,76666667** | **41,93333** | **39,16666667** | **8,533333333** | **10,1** |
| SEM | 4,167067 | 4,643753271 | 4,267838 | 2,983472995 | 1,010500426 | 1,946792233 |

**Table 9**

**Figure 5 H** (MTT assay of HeLa cells after knockdown of SURF6)

|  | MTT HeLa |  |  |  |  |  |
| --- | --- | --- | --- | --- | --- | --- |
|  | Control |  |  | knockdown |  |  |
|  | 24 h | 48 h | 72 h | 24 h | 48 h | 72 h |
|  | 0,354 | 0,493 | 0,777 | 0,403 | 0,606 | 0,806 |
|  | 0,387 | 0,575 | 0,74 | 0,387 | 0,579 | 0,761 |
|  | 0,395 | 0,581 | 0,778 | 0,39 | 0,597 | 0,796 |
|  | 0,378 | 0,596 | 0,776 | 0,368 | 0,583 | 0,771 |
|  | 0,403 | 0,554 | 0,765 | 0,397 | 0,606 | 0,765 |
|  | 0,372 | 0,565 | 0,7 | 0,387 | 0,621 | 0,767 |
|  |  |  |  |  |  |  |
| mean | 0,3815 | 0,560667 | 0,756 | 0,388667 | 0,598667 | 0,777667 |
|  |  |  |  |  |  |  |
| SEM | 0,00715 | 0,01473 | 0,012646 | 0,004863 | 0,006433 | 0,007606 |

**Table 10**

**Figure6c,D,E, F,G** (HCT116 p53+ and HCT116 p53- cell cycle analysis after knockdown of SURF6)

|  | P53+ SURF6+ | | | P53+ SURF6- | | |
| --- | --- | --- | --- | --- | --- | --- |
|  | G1/G0,% | S ,% | G2/M,% | G1/G0,% | S ,% | G2/M,% |
|  |  |  |  |  |  |  |
|  | 50,4 | 23,8 | 25,8 | 53,4 | 22,6 | 24 |
|  | 51 | 22 | 27 | 58,2 | 16,1 | 25,7 |
|  | 57,6 | 18,8 | 23,6 | 60,3 | 18 | 21,7 |
|  | 56,6 | 20,4 | 23 | 60,7 | 18,1 | 21,2 |
| Mean | 53,9 | 21,25 | 24,85 | 58,15 | 18,7 | 23,15 |
| SEM | 1,862793601 | 1,071992 | 0,93586 | 1,67556 | 1,379009 | 1,046024 |

|  | P53- SURF6+ | | | P53- SURF6- | | |
| --- | --- | --- | --- | --- | --- | --- |
|  | G1/G0,% | S ,% | G2/M,% | G1/G0,% | S ,% | G2/M,% |
|  |  |  |  |  |  |  |
|  | 47,9 | 19,1 | 33 | 50,8 | 19,2 | 30 |
|  | 50,6 | 17,1 | 32,3 | 48,9 | 22,2 | 28,9 |
|  | 44 | 24,8 | 31,2 | 51,9 | 23,6 | 24,5 |
|  | 40,2 | 24,1 | 35,7 | 50,1 | 24,6 | 25,3 |
| Mean | 45,675 | 21,275 | 33,05 | 50,425 | 22,4 | 27,175 |
| SEM | 2,272 | 1,883 | 0,958 | 0,628 | 1,175 | 1,342 |

**Table 11**

**Figure 6 H** (MTT assay of HCT116 p53+ and HCT116 p53- after knockdown of SURF6)

| MTT HTC116 | P53+ SURF6- | P53+ SURF6+ | P53- SURF6- | P53- SURF6+ |
| --- | --- | --- | --- | --- |
|  | 1,225 | 0,714 | 1,25 | 0,74 |
|  | 1,174 | 0,9 | 1,05 | 0,76 |
|  | 1,179 | 0,93 | 1,116 | 0,737 |
| Mean | 1,192 | 0,848 | 1,139 | 0,746 |
| SEM | 0,016 | 0,069 | 0,059 | 0,007 |

**Table 12**

**Figure 6 A,B** (western blot analysis)

| Sample | [P53/actin]_si_ | [P53/actin]_ctr_ | [P53/actin]_si_/[P53/actin]_ctr_ |
| --- | --- | --- | --- |
| 1 | 0,42 | 0,31 | 1,418 |
| 2 | 0,81 | 0,83 | 0,98 |
| 3 | 0,48 | 0,63 | 0,76 |
| 4(Fig) | 0,68 | 0,61 | 1,09 |
| Mean | 0,6 | 0,6 | 1,06 |
| SEM | 0,09 | 0,11 | 0,14 |
