# Supplementary material for: Human nucleolar protein SURF6/RRP14 participates in early steps of pre-rRNA processing
Source: PLoS One. 2023 Jul 14;18(7):e0285833. doi: 10.1371/journal.pone.0285833 (PMC10348582; doi:10.1371/journal.pone.0285833)
Supplement: S1 Raw images — (PDF) [file pone.0285833.s003.pdf]

Raw_Fig. 1

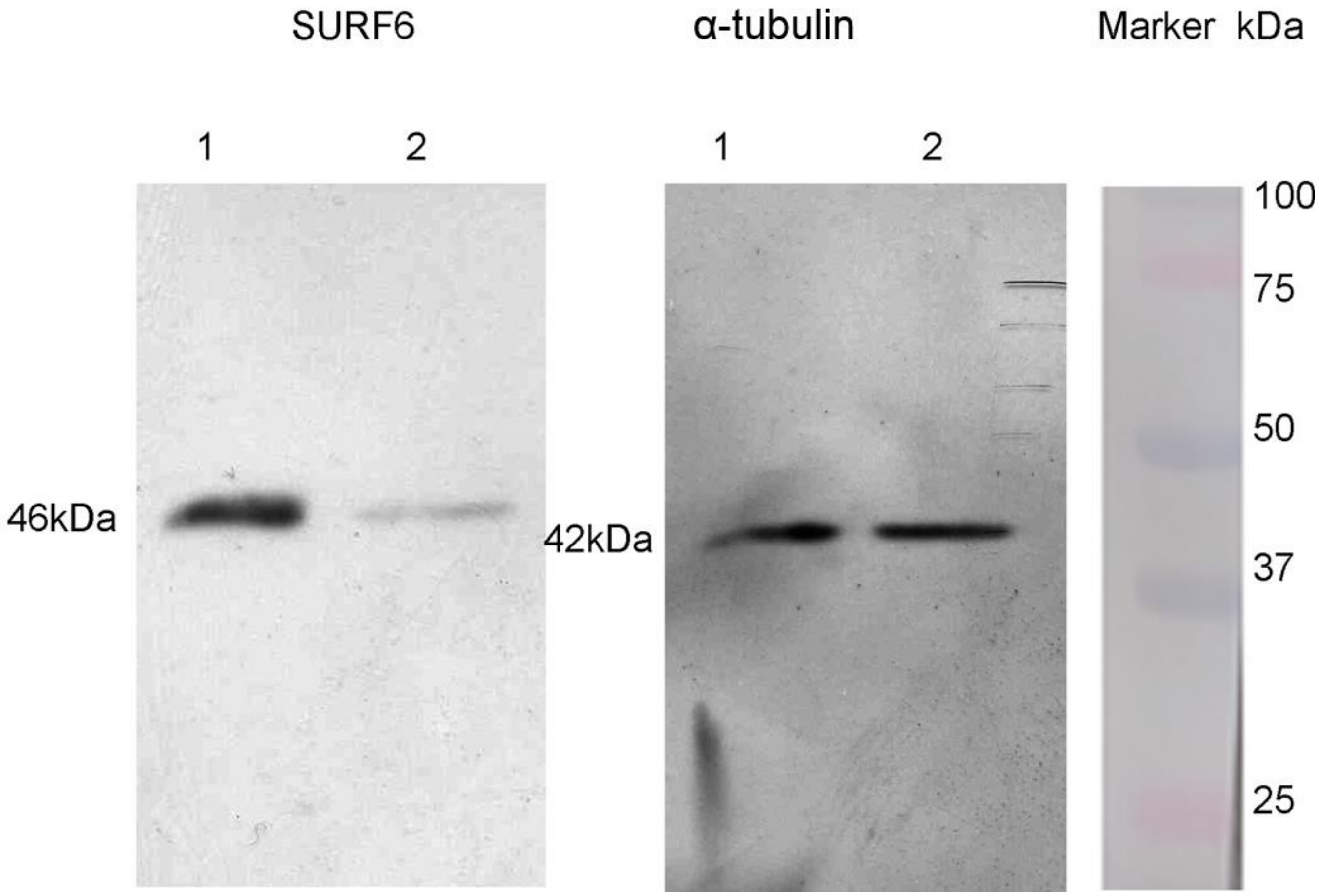

Raw_fig. 1 description: western blot data from Raw_fig. 1 were obtained using X-ray film and were used for the preparation of Fig 1A panel in the manuscript. Lanes labeled “1” on both blots represent protein samples from cells transfected with non-targeting siRNA. Lanes labeled “2” on both blots represent protein samples from cells with si-mediated SURF6 knockdown.

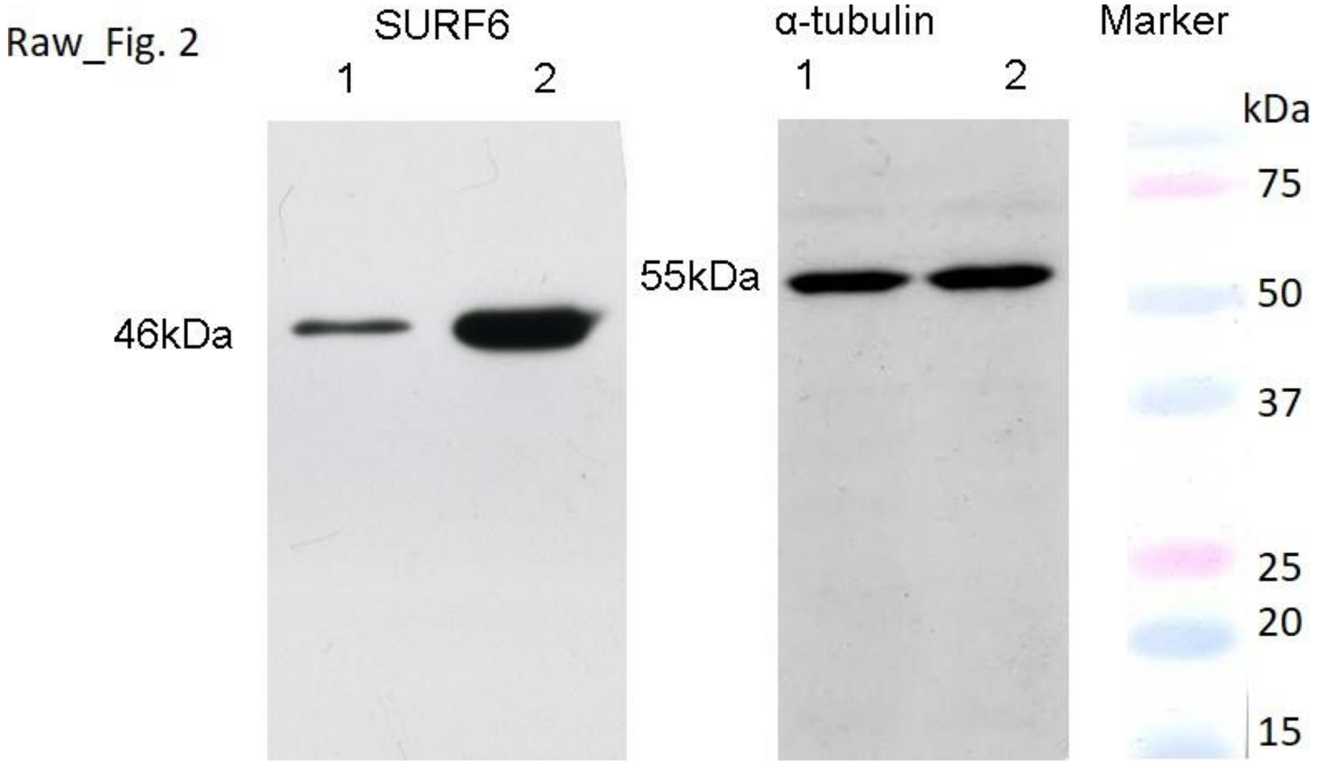

Raw_fig. 2 description: western blot data from raw_fig. 2 were obtained using on the X-ray film and were used for the preparation of Fig 2A panel in the manuscript. Lanes labeled “1” on both blots represent protein samples from cells treated with non-modified pCDNA3.1 vector. Lanes labeled “2” on both blots represent protein samples from cells transfected with pCDNA3.1-hSURF6 for human SURF6 overexpression.

Raw_fig. 3

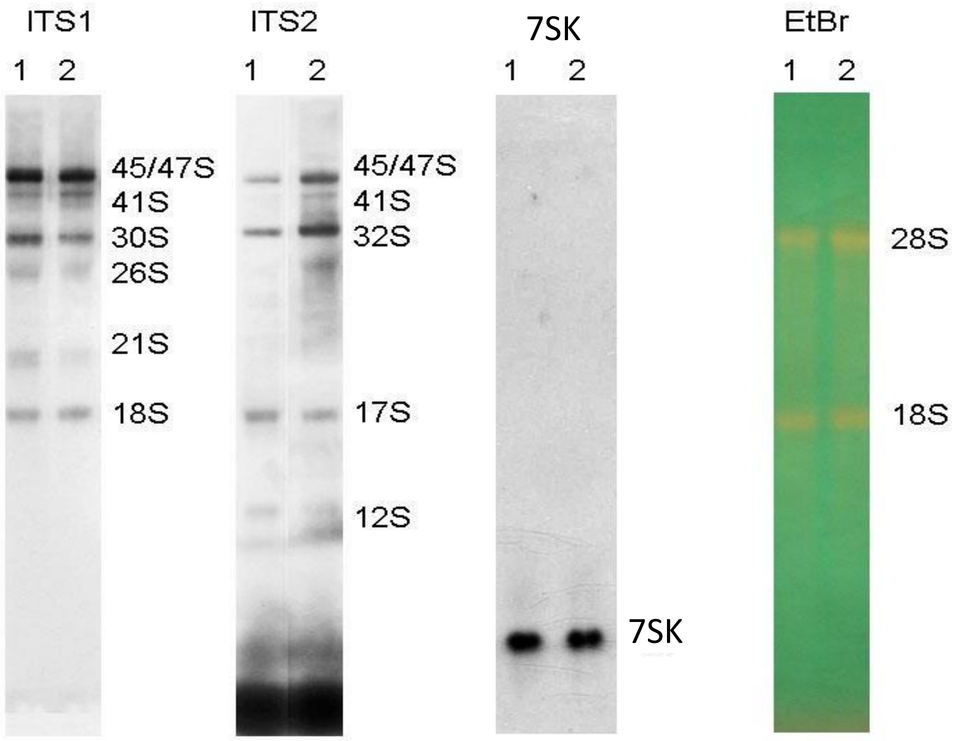

Raw_Fig.6

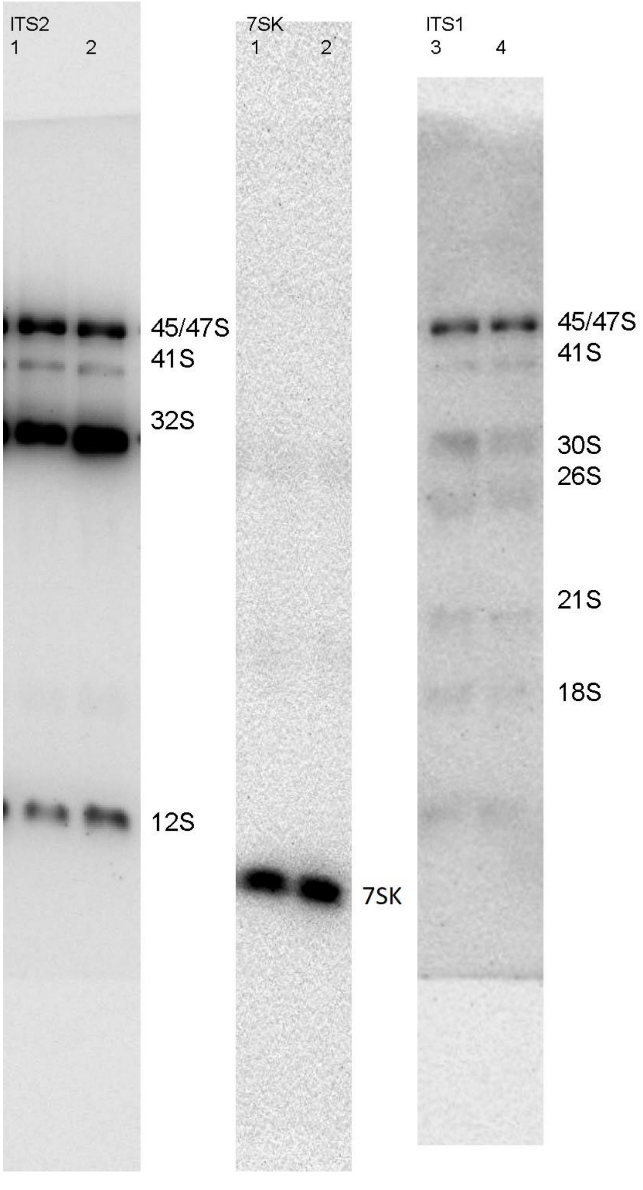

Raw_Fig.8

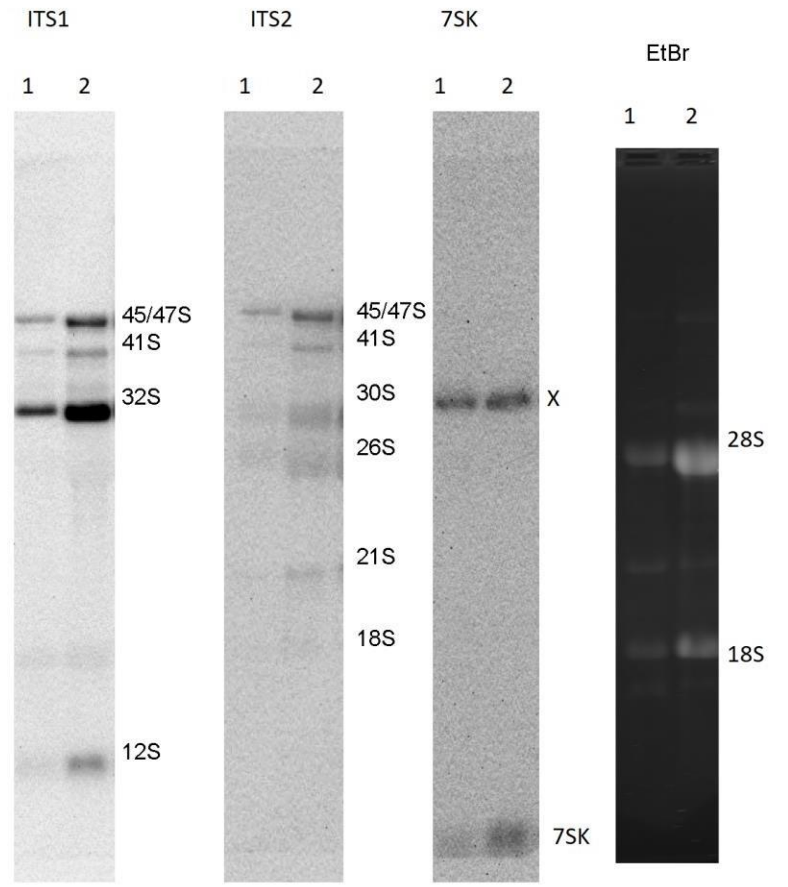

<u>Raw_fig. 3, raw_fig. 6, raw_fig. 8 description:</u> northern blot data from Raw_fig. 3 were obtained using X-ray film, data on raw_fig. 6 and raw_fig. 8 were obtained using ChemiDoc Imaging System (BioRad). All raw figures represent northers blotting experiment made in triplicates. Cropped variants of raw_fig. 6 were used to prepare Fig. 4 B, E panels in the manuscript. Lanes labeled “1” on blots represent protein samples from cells treated with non-modified pCDNA3.1 vector. Lanes labeled “2” on both blots represent protein samples from cells transfected with pCDNA3.1-hSURF6 for human SURF6 overexpression. Non-specific signals are labeled “X”.

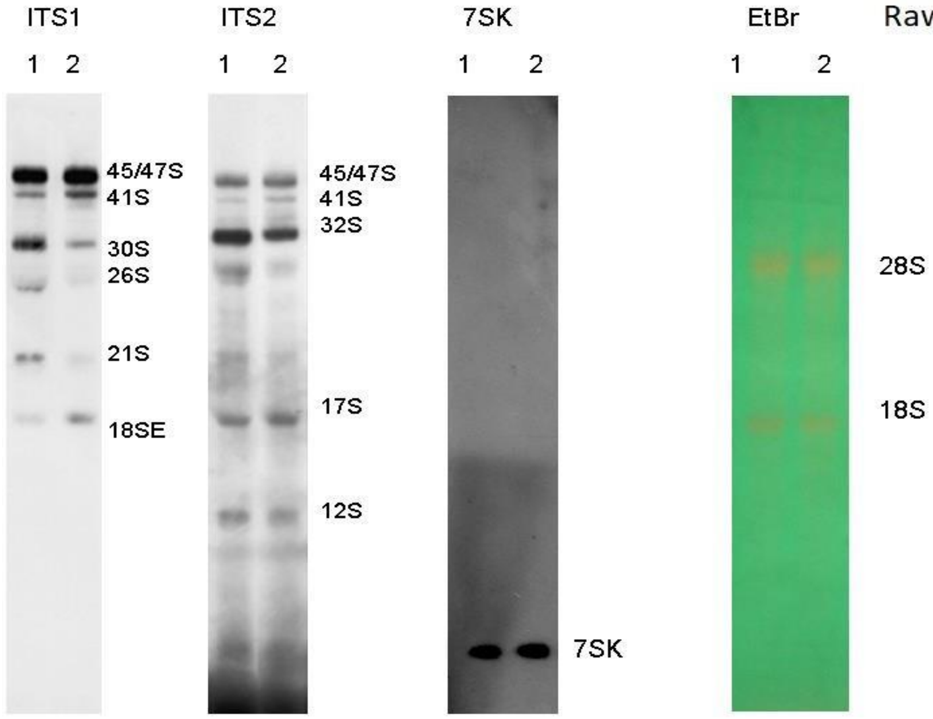

Raw_Fig.4

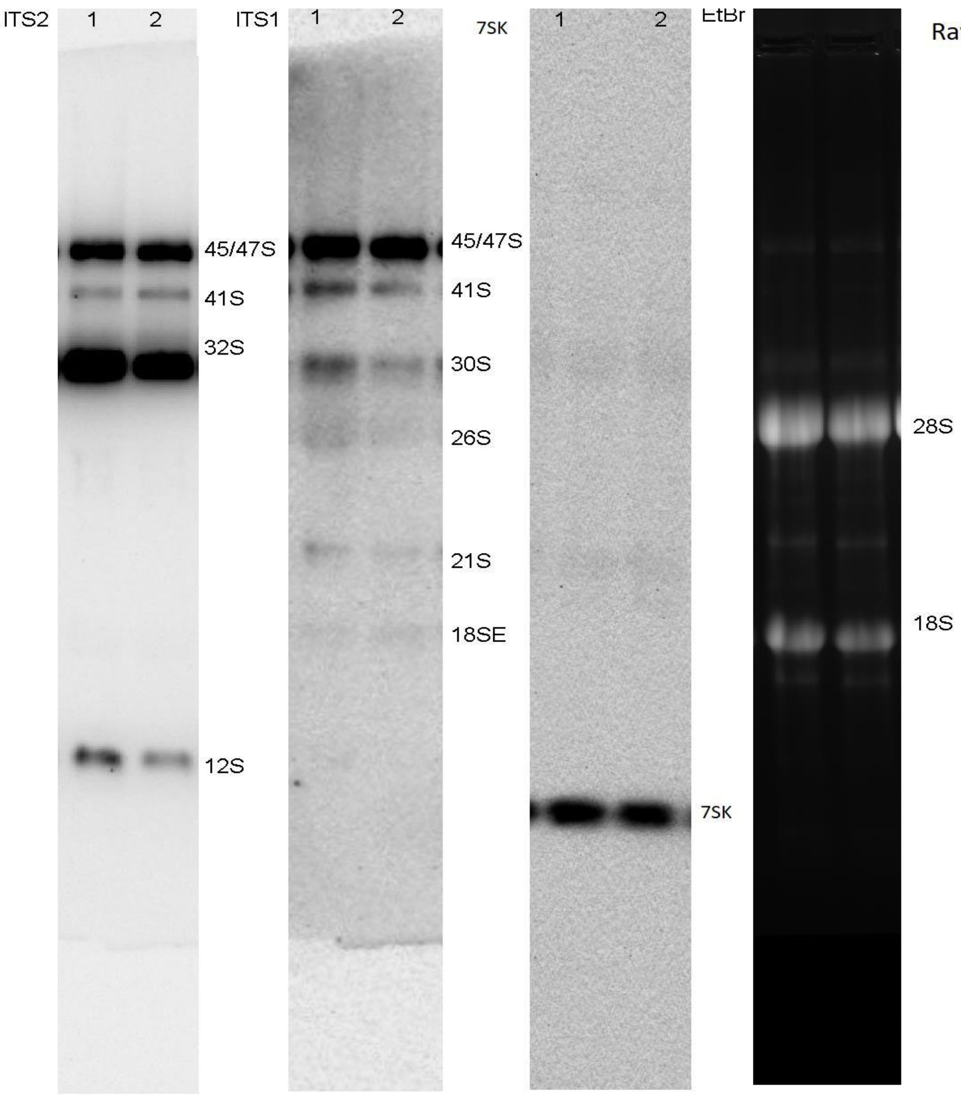

Raw_Fig. 5

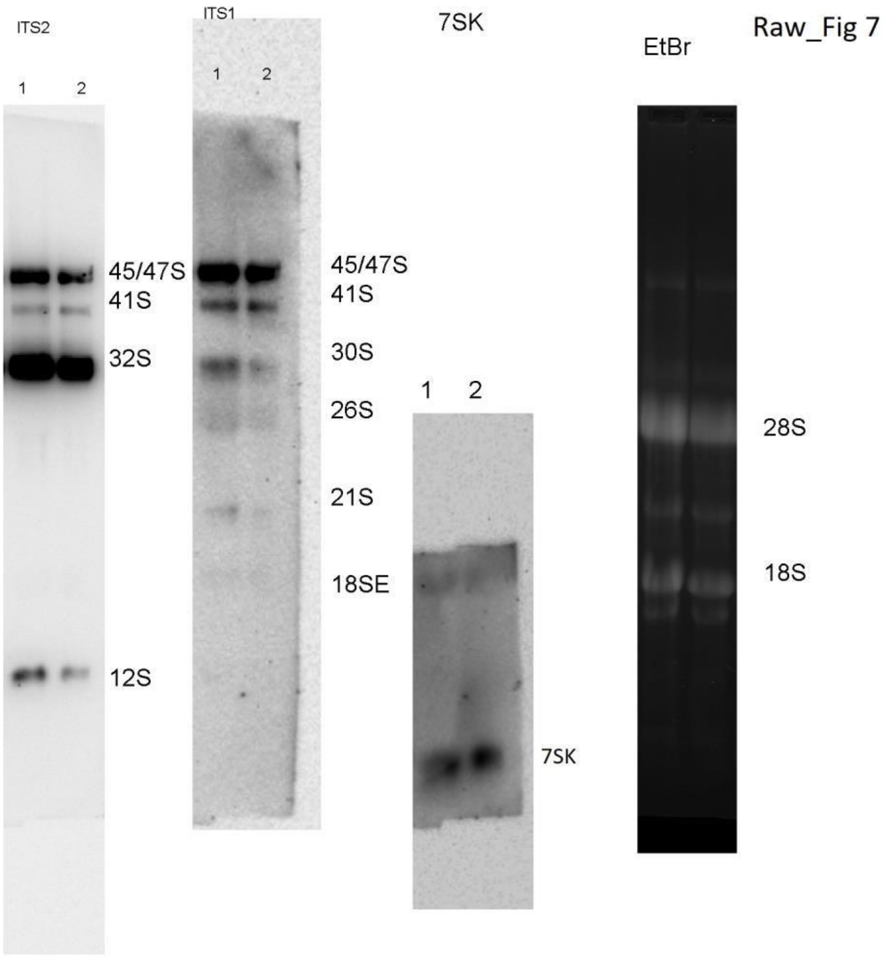

<u>Raw_fig. 4, raw_fig. 5, raw_fig. 7 description:</u> northern blot data from Raw_fig. 4 were obtained using X-ray film, data on raw_fig. 5 and raw_fig. 7 were obtained using ChemiDoc Imaging System (BioRad) and were used to prepare Fig 4 A, D panels in the manuscript. Lanes labeled “1” on blots represent protein samples from cells treated with non-targeting siRNA. Lanes labeled “2” on both blots represent protein samples from cells si-mediated knockdown.

Raw_Fig.9

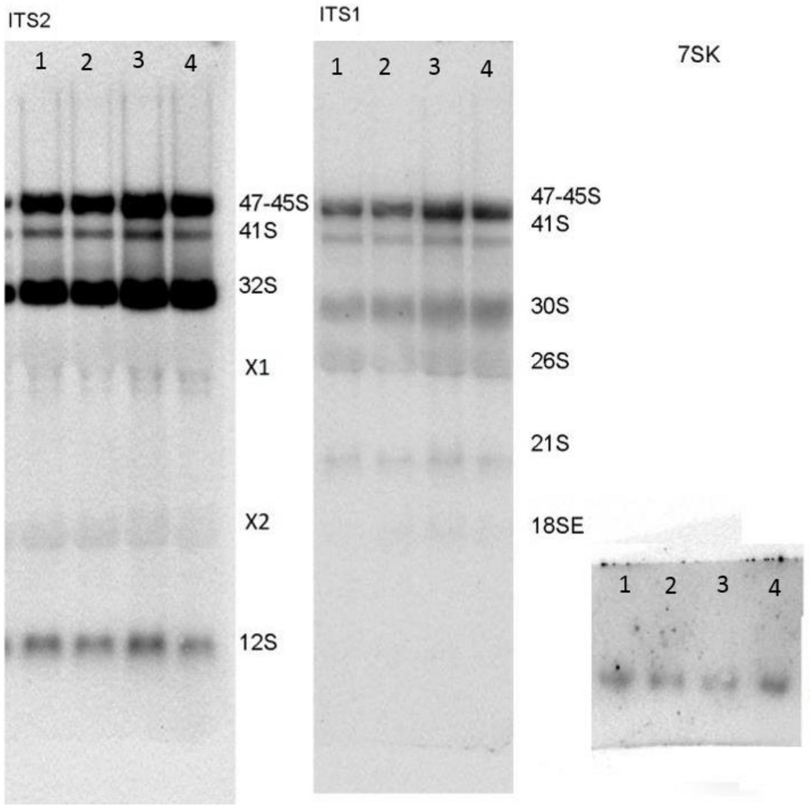

Raw_Fig.10

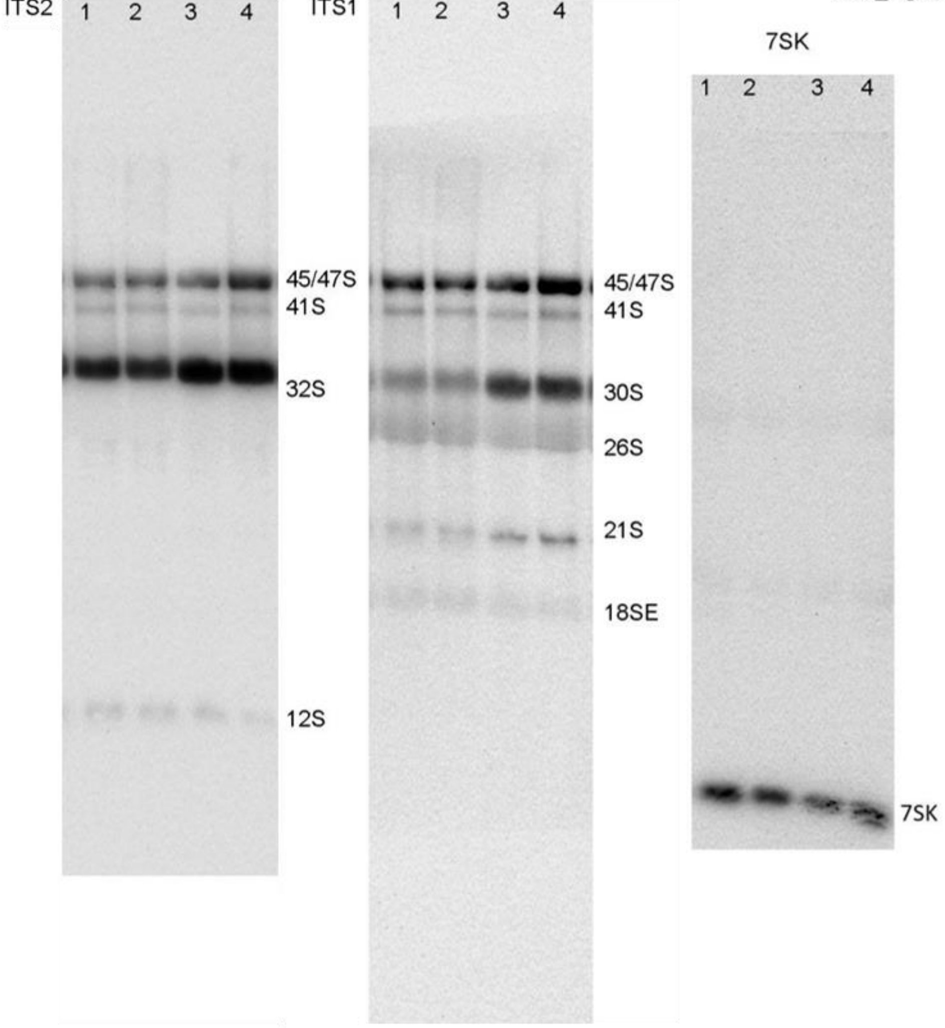

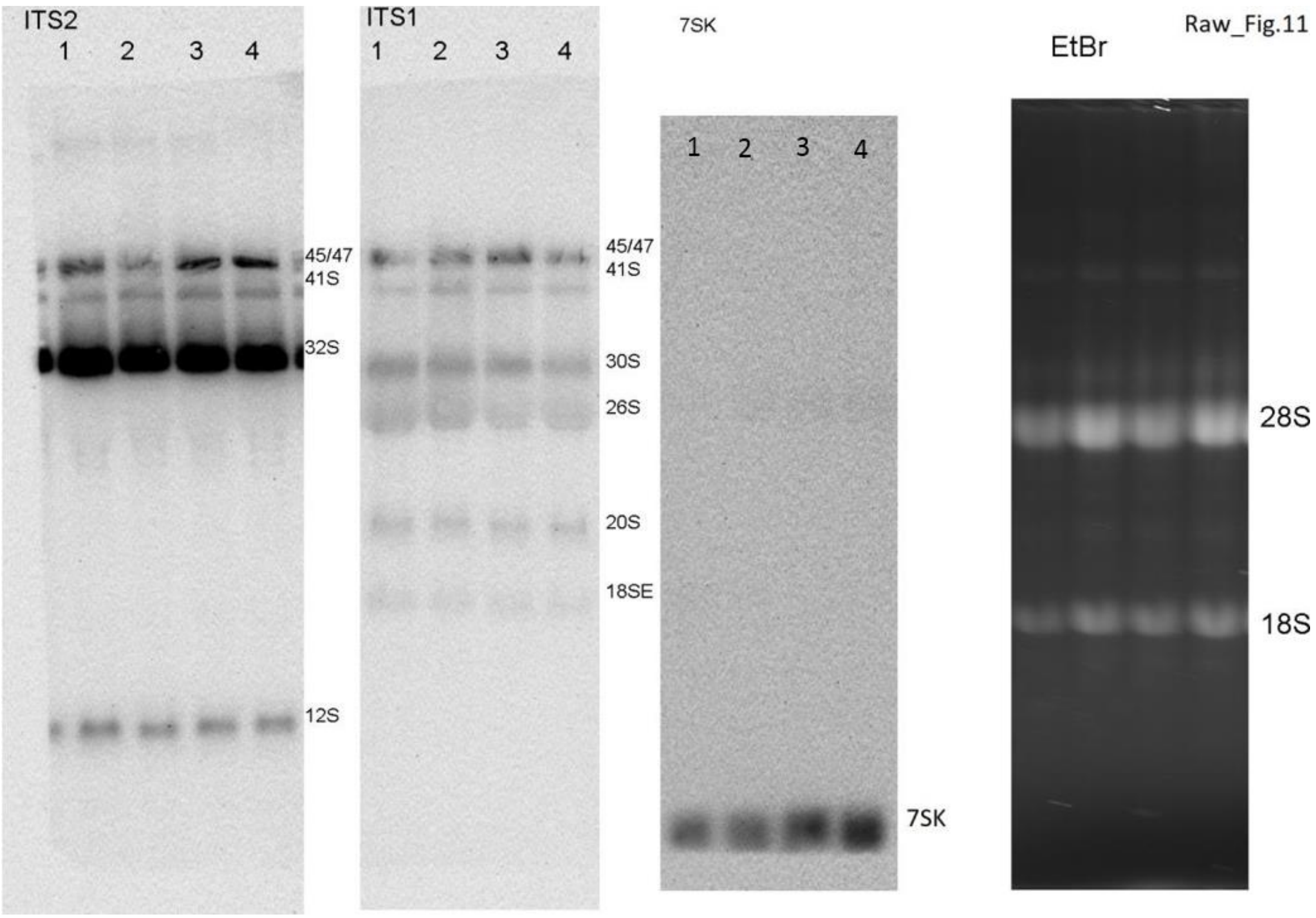

Raw_fig. 9, raw_fig. 10, raw_fig. 11 description: northern blots from raw_fig. 9-11 represent experiment made in triplicates. Data from raw_fig. 9 were used to prepare Fig 4 F panel, while dad from raw_fig. 10 were used to prepare Fig 4 C panel. Signals after EtBr staining or oligos hybridization were obtained using ChemiDoc Imaging System (BioRad). On all panels samples were loaded in the following order: 1 – HCT116 p53- SURF6+, HCT116 p53- SURF6-, 3 – HCT116 p53+ SURF6+, 4 – HCT116 p53+ SURF6-. Non-specific signals on raw_fig. 9 are labeled "X1, X2".

Raw_Fig.12

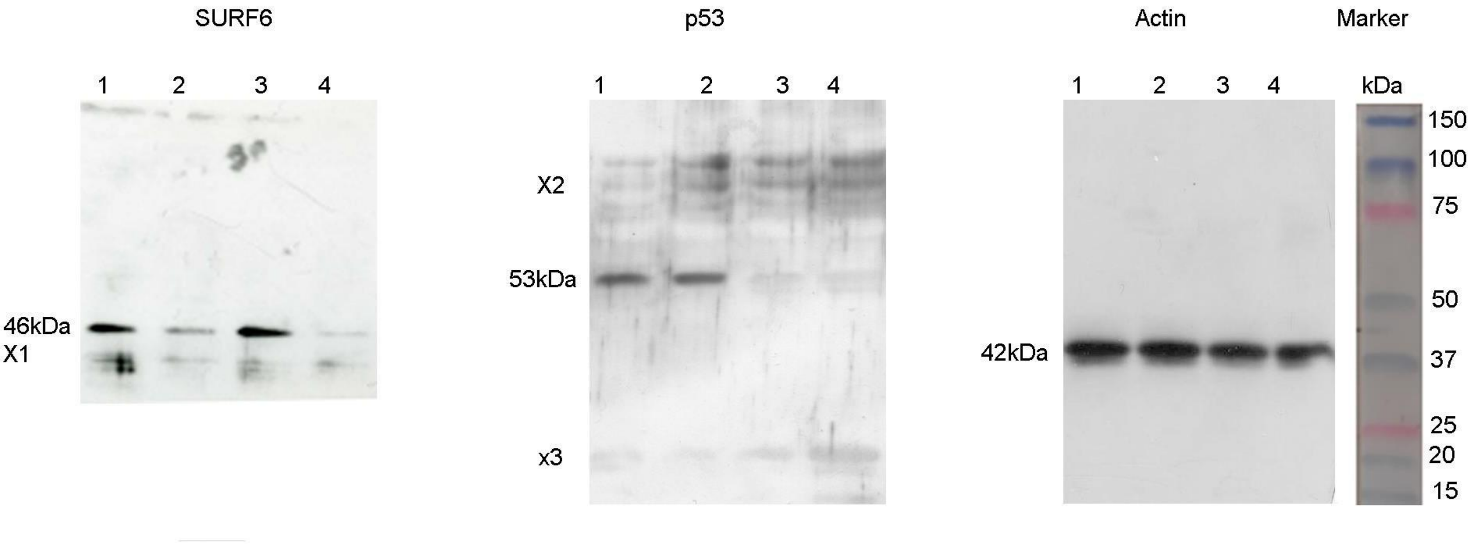

Raw_fig. 12 description: western blot data from raw_fig.12 were produced on the X-ray film and were used to prepare Fig 6A panel in the manuscript. On all panels samples were loaded in the following order: 1 – HCT116 p53+ SURF6+ (non-targeting siRNA), 2 – HCT116 p53+ SURF6- (anti-SURF6 siRNA), 3 – HCT116 p53- SURF6+ (non-targeting siRNA), 4 – HCT116 p53- SURF6- (anti-SURF6 siRNA). Non-specific signals are labeled "X1, X2, X3".

Raw_fig.13

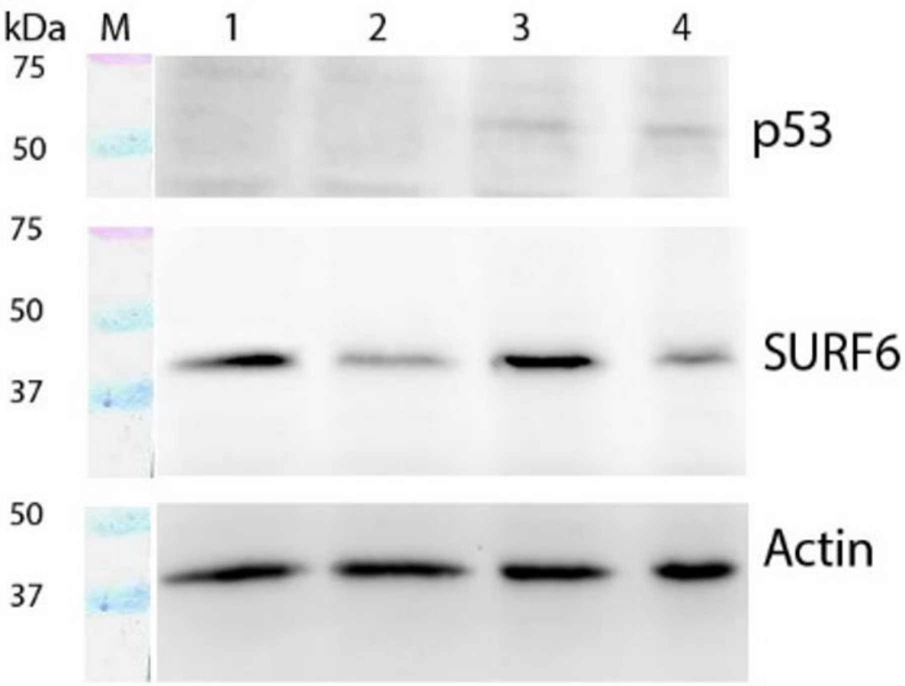

Raw_fig.14

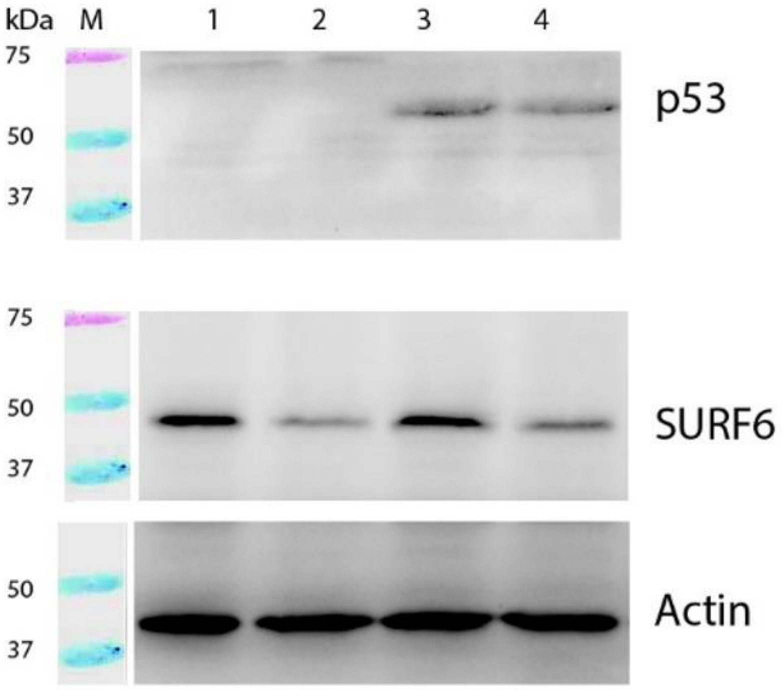

Raw_fig.15

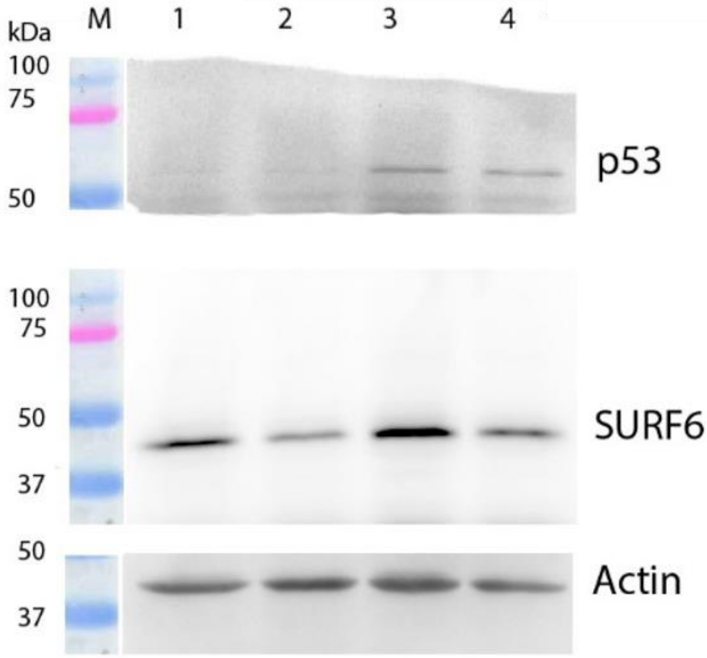

<u>Raw_fig. 13, raw_fig. 14, raw_fig. 15 description:</u> western blot data from raw_fig. 13 and 14 were obtained using ChemiDoc Imaging System (BioRad). Data on raw figures 12, 13, 14 and 15 represent Surf6 knockdown experiments on HCT116 p53+ or p53- cells made in four replications. On all panels samples were loaded in the following order: 1 – HCT116 p53- SURF6+ (non-targeting siRNA), 2 – HCT116 p53- SURF6- (anti-SURF6 siRNA), 3 – HCT116 p53+ SURF6+ (non-targeting siRNA), 4 – HCT116 p53+ SURF6- (anti-SURF6 siRNA).
