# Supplementary material for: Human nucleolar protein SURF6/RRP14 participates in early steps of pre-rRNA processing
Source: PLoS One. 2023 Jul 14;18(7):e0285833. doi: 10.1371/journal.pone.0285833 (PMC10348582; doi:10.1371/journal.pone.0285833)
Supplement: Attachment — Submitted filename: Response to Reviewers Remarks to the Authors.docx [file pone.0285833.s004.docx]

Rebuttal letter for manuscript

PONE-D-22-03434

Moraleva et al. Human nucleolar protein SURF6/RRP14 participates in early steps of pre-rRNA processing

PLOS ONE

Dear
Dr. Jorge Perez-Fernandez,

First, my colleagues and I would like to thank you and both reviewers for careful and professional evaluation of our manuscript. We are pleased to receive your kind remarks concerning the quality of data and scientific value of observations described in our study. The issues and concerns raised by the reviewers are reasonable, and it took time and effort to perform some extra experiments and analysis to answer most (in our opinion), if not all, points. The revised version of the manuscript is greatly improved and not only shows the involvement of SURF6 to processing of pre-rRNA, but also provides hints concerning the changes in cell cycle caused by overexpression or downmodulation of SURF6 in human cells.

Please, find our point-by-point response to reviewers below.

Reviewers’ comments are shown in italic. Our response is in straight font. Changes in the main text of the manuscript are labeled yellow.

***Reviewer #1:*** *In their manuscript, the authors characterize the role of Surf6 on cell growth and ribosome biogenesis in the Hela cell line. Surf6 is the homolog of the well-characterized yeast factor Rrp14, which is involved in the early stages of ribosome assembly. The authors and other researchers have studied Surf6 in mice and humans. The results available in the bibliography support a functional conservation between Surf6 and Rrp14, and also indicate that Surf6 is a growth factor. Recently, Surf6 was characterized as a direct partner of Nucleophosmin (NPM1), a component of the nucleolus, suggesting that Surf6 is also present in this sub-nucleolar compartment.*

*To fully characterize the function of Surf6 in humans, the authors analyzed its localization by immunofluorescence experiments and confirmed its nucleolar accumulation. Then, they studied the effect of its knockdown (KD) and overexpression (OE) on pre-rRNA processing and confirmed a functional conservation between Rrp14 and Surf6 on pre-rRNA processing. Finally, they evaluated the effect of its depletion and overproduction on the cell cycle by flow cytometric analysis.*

*First of all, I would like to emphasize that the figures are of good quality. However, several points lead to question the overall significance of the work and fail to convince that this manuscript is worth publishing.*

We appreciate the effort made by the reviewer to judge our study. And happy to provide detailed response to his comments:

*My main comments are as follows:*

*- The authors analyzed the co-localization of Surf6 (by an immuno-fluorescence (IF) approach) with different pre-rRNA precursors (ITS1, ITS2, and 18S) probed by FISH (Figure 1). This method, using fluorescence, is not resolutive enough to conclude on which pre-rRNAs Surf6 is best co-localized. Electron microsocpy would be the only tool to access to such data. Furthermore, the reduced co-localization of Surf6 with 18S and 28S does not make sense since mature 18S and 28S rRNAs accumulate mainly in the cytoplasm. This entire section simply serves to confirm that Surf6 is primarily accumulated in the nucleolus, and is therefore redundant with Figure 2.*

We agree with the reviewer that combination (overlay) of FISH for ITS1, ITS2, 18S and 28S probes to pre-rRNA fragments and IF for SURF6 is not the best way to answer the question concerning co-localization of SURF6 with the regions of nucleolus involved in biogenesis of rRNA precursors. We had in mind that we will be not get definitive answers concerning precise co-localization of SURF6 with areas where particular rRNA precursors reside. But these data gave us a clue and indicated that in normal HeLa cells SURF6 localization significantly overlaps with areas where these precursors could be found. Therefore, Fig. 1 shows simply that it’s reasonable to imply that SURF6 in HeLa cells participates in early steps of the rRNA biogenesis. Concerning 18S and 28S, it is true that majority of these RNAs are localized in the cytoplasm, but in Fig.1 we show their localization as parts of larger precursors. We changed Fig. 1D to make clearer where corresponding FISH probes anneal in full length pre-rRNA.

Figure 2 was changed significantly (see figures and figure legends) by adding confocal microscopy of cells co-stained with SURF6 and nucleolar markers that label functional zones of nucleolus, fibrillarin and B23. Therefore, Fig. 1 and new Fig. 2 lack redundancy noted by reviewer.

Significant part of the text was modified and is shown below (lines 249 – 257 of revised text):

In our previous study we demonstrated that murine Surf6 is involved in ribosome biogenesis, which was not shown for human SURF6. Therefore, we decided to study co-localization of SURF6 with ITS1, ITS2, 18S and 28S parts of pre-rRNA in nucleoli of fixed/permeabilized HeLa cells using FISH with respective specific probes (Fig. 1N, scheme of 47S pre-rRNA and location of probes) and immunostaining of SURF6 (Fig. 1). The goal of this experiment was to determine at what stage (early or late) of pre-rRNA processing SURF6 might participate. Despite an imperfection of this approach, it is clear from confocal images that signal from SURF6 almost completely overlaps inside the nucleoli with the signal from ITS2 FISH (Fig. 1D, E, F). At the same time, SURF6 also significantly co-localizes with the areas of ITS1 FISH signal (about 70% overlap, Fig. 1A, B, C). Overlay of SURF6 immunostaining with FISH of 18S and 28S was less prominent (Fig. 1G-L).

*- In Figure 2, the authors compare the effect of Surf6 depletion and overexpression on cell morphology. To do so, they perform IF against Surf6 and obtained a nucleolar signal (co-localization with nucleolus marker is missing) that is stronger when Surf6 is overexpressed, but is absent after its depletion, confirming the good depletion conditions. Then, to assess the effect of its EO and KD on cell morphology, they use DAPI staining and phase contrast. These two methods are not appropriate for testing the change in cell morphology associated with ribosomal assembly defects. Indeed, examination of nucleolus staining would be much more informative and the only way to conclude on the effect of alterations in Surf6 expression on cell morphology. I would invite the authors to perform such analysis by probing the nucleolus either by FISH (ITS1) or by using the IF against classical nucleolus markers (NPM1, fibrillarin etc...).*

We would like to agree with the concern of the reviewer. To solve the issue, we performed confocal microscopy of fixed control HeLa cells and cells with SURF6 knockdown that were triple stained with DAPI, anti-SURF6, and anti-fibrillarin (fibrillar nucleolar component marker) or anti-B23 (granular nucleolar component marker). One can see (Fig. 2, D, H, N, R) that morphology of fibrillar component and global architecture of the nucleolus is not affected by the SURF6 KD.

Following text was added to the manuscript (lines 280-285 of revised text):

To examine potential changes in fine architecture of nucleolus, we co-stained nucleoli of control HeLa and SURF6 knockdown HeLa with anti-SURF6 and anti-fibrillarin (nucleolar fibrillar component marker) or anti-SURF6 and anti-B23 (nucleolar granular component marker) (Fig. 2C-J, M-T). Confocal images demonstrate that fine patterning of fibrillarin and B23 are not affected by SURF6 knockdown. It is interesting that fibrillarin signal is more punctate than that of SURF6 suggesting that SURF6 could potentially function outside fibrillar centers.

In case of overexpression, we transfected HeLa cells with SURF6-GFP fusion construct and stained them with DAPI and the probe to ITS1. Corresponding supplemental figure (see Suppl. Fig. 2) demonstrates that morphology of the nucleolus is not visibly affected by overexpression of SURF6-GFP chimera.

Please, see changes in the text (lines 295-300 of revised text):

Further, we stained cells that overexpress SURF6-GFP fusion with ITS1 FISH probe to demonstrate that fine structure of nucleolus is not affected as well (Suppl. Fig. 2). These results suppose that overexpression of human SURF6 in HeLa does not disturb cell morphology similar to the consequence of SURF6 overexpression in mouse embryonic cells [11]. Formally, it is not possible to exclude that there are faint changes in nucleoli of HeLa cells following overexpression or knockdown of SURF6, but they would require complicated high resolution EM studies.

*- Finally, the authors analyzed the effect of Surf6 OE and KD on the cell cycle (Figure 4). When ribosome assembly is affected, a nucleolar stress response pathway is activated and leads to p53 accumulation. This generally promotes cell cycle arrest in G1 and even apoptosis if this stress is maintained. Here, in stark contrast to what the authors observe in Figure 3 supporting a ribosome assembly defect, no G1 arrest is observed, only a longer S phase could be noted.
These unusual results should be clarified and discussed in the text. For example, is p53 expression affected under these conditions, we know that lipofectamine transfection clearly plays a role on p53 activation by itself, which could disrupt subsequent cell cycle analysis. Another possibility to interpret this lack of G1 arrest after ribosomal assembly defects would be a direct role of Surf6 in the regulation of p53 in response to nucleolar stress. These results require further scrutiny before publication, as there are many elements that may impact cell cycle analysis.*

Again, we would like to thank the reviewer for asking this important question.

First, for HeLa cell cycle in SURF6 WT/KD, we added MTT test data that show statistically insignificant but detectable increase in viability of SURF6 KD cells in comparison to WT. This indicates, although not formally proves, that SURF6 KD does not decrease survival of cells.

Please, see text (lines 380-383 of revised text):

The analysis of cell cycle itself could be misleading without evaluation of cell viability/proliferation. To close this gap, we performed MTT-test to compare viability/proliferation of SURF6 knockdown and control HeLa cells (Fig. 4H). In striking contrast to murine fibroblasts, SURF6 knockdown has no negative effect on cell proliferation of HeLa cells.

Second, for some reason HeLa cells have very short G2 phase (Poudyal D, Herman A, Adelsberger JW, Yang J, Hu X, Chen Q, Bosche M, Sherman BT, Imamichi T. A novel microRNA, hsa-miR-6852 differentially regulated by Interleukin-27 induces necrosis in cervical cancer cells by downregulating the FoxM1 expression. Sci Rep. 2018 Jan 17;8(1):900. doi: 10.1038/s41598-018-19259-4.) and lack expression of functional p53 protein (doi:10.1101/gad.9.17.2170Genes & Dev. 1995. 9: 2170-2183).

Therefore, we tend to speculate that this abnormal cell cycle and loss of control from p53 in WT HeLa affects the changes in cell cycle distribution of HeLa with SURF6 KD/OE. It should be also stressed out that despite very significant changes in pre-rRNA processing we do not see decreased viability of cells by MTT.

Corresponding text (lines 384-387 of revised text):

HeLa cell line has defect in p53 expression [26]. It has been shown that they express p53 mRNA, which is not translated, therefore ribosomal stress in HeLa caused by disturbance of pre-rRNA processing possibly would not lead to arrest in G1 phase and subsequent apoptosis. Meanwhile, in human cells with normal p53 expression one would expect negative effect of SURF6 knockdown on cell proliferation and viability.

Third, according to the literature, there is a link between p53 and ribosome biogenesis/ribosomal stress. In human cancer cells the control of cell cycle from p53 is often lost which means that data obtained in cancer cell lines could be affected by this defect. To address this point, we set up SURF6 siRNA transient KD experiments in HCT116 p53+ and HCT116 p53- cell lines. HCT116 p53+ cell line has normal regulation of p53 activity. The results of cell cycle distribution and MTT test are presented in Fig.5 which we added to the manuscript. Fig.5A demonstrates efficiency of siRNA mediated KD in both cell lines. Analysis of cell cycle distribution (Fig.5B, C) clearly indicates that, independent on the presence of p53, SURF6 KD increases proportion of cells in G0/G1 and decreases in G2/M phase. S phase does not change significantly. This result is in the striking contrast with HeLa cells where we see effect on S phase. Viability of cells in both p53- and p53+ cells go up in the case of SURF6 KD (Fig.5D). From these results we concluded that SURF6-mediated effects on cell cycle are independent on p53, despite dramatic effects on pre-rRNA processing. This raises the possibility that SURF6 may have two distinct functions, or that somehow deficiency in SURF6 and ribosome biogenesis defects are uncoupled from the p53 regulation of cell cycle in HCT116 cells. This unexpected phenomenon deserves further study.

We added section to the manuscript (lines 388-399 of revised text):

**SURF6 knockdown affects the cell cycle in HCT116 cell line in p53-independent manner**

The question regarding a role of p53 in SURF6 effects on the cell cycle could be answered by direct comparison of cell cycle and proliferation/viability of p53 sufficient and p53 deficient cells with SURF6 knockdown. We used a pair of HCT116 cell lines which differ only in expression of the p53 protein (and designated HCT116 p53+ and HCT p53-, respectively). siRNA-mediated knockdown of SURF6 was induced in both cell lines by transient transfection with corresponding control and SURF6-specific siRNA (Fig. 5A). Then we compared the cell cycle distribution of control and SURF6 knockdown cells expressing or lacking p53. In both p53+ and p53- HCT116 SURF6 knockdown caused statistically significant increase in proportion of cells in G0/G1 phase, had no effect on S phase and markedly decreased G2/M phase (Fig. 5B-F). According to MTT-test, proliferation/survival of HCT116 p53+ or p53- cell lines was not negatively influenced by SURF6 knockdown (Fig. 5G). Based on this data, we suggest that SURF6 affects cell cycle in HCT116 cell line largely in p53-independent manner.

*Minor comments:*

*-Figure 3, In the RAMP analysis €"18S" should be replaced by 18SE. In the human precursor of 18SE, it is 21S, please also check the labeling in panel (E).*

We thank reviewer for pointing to the mistake.

Changes were made in the Fig. 3E, 18S was replaced by 18SE.

*-The comments on Figure 4 are missing in the discussion section.*

We thank the reviewer for this comment. The discussion section was updated according to the data from figures 4 and 5 (lines 455-461 of revised text):

Study of cell cycle distribution changes caused by SURF6 knockdown in HeLa revealed surprising results. Unlike murine fibroblasts or primary human cells, HeLa did not suffer from G1 phase arrest and subsequent apoptosis. Instead, they proliferated as good as control HeLa and had unexpected increase in S phase (Fig. 4A-D). These strange phenotypes could be explained by the defect in p53 expression in HeLa cells [26]. They lack functional p53 protein due to translational incompetence of the p53 mRNA. In this regard, we tested the effect of SURF6 knockdown in variants of HCT116 human cell line one of which has normal regulation and amount of p53, while another was modified to induce p53 knockout [13].

*Overall, I think the manuscript suffers from two major flaws: (1) Lack of nucleolar straining after both Surf6 overexpression and knockdown (2) Necessity to perform additonal controls to properly interpret absence of G1 arrest after Surf6 depletion.*

We fixed both major flaws by making additional experiments and providing data (please, see response to major points and Fig. 2D, H, N, R, Suppl. Fig. 2, Fig. 4H, and Fig.5). Text of the manuscript was changed accordingly.

***Reviewer #2:*** *In this manuscript, Moraleva et al. investigate the importance of human SURF6 in ribosome biogenesis and cell cycle progression. Using a combination of co-localisation studies, pre-ribosomal RNA analysis and flow cytometry in HeLa cells, they found that knockdown (KD) or overexpression (OE) of SURF6 affects ribosome production (by switching pre-rRNA processing to an alternate pathway) and also has a mild impact on the cell cycle, while, in contrast to previous studies in embryonic fibroblasts, this does not result in cellular death.
While the presented data are of interest, in particular the novel observation that SURF6 might be needed for site 2 pre-rRNA cleavage in human cells, the advances compared to one of their previously published studies in mouse cells are not that obvious (Anastasiia Moraleva, Charalambos Magoulas, Mikhail Polzikov, Sabine Hacot, Hichem C. Mertani, Jean-Jacques Diaz & Olga Zatsepina (2017) Involvement of the specific nucleolar protein SURF6 in regulation of proliferation and ribosome biogenesis in mouse NIH/3T3 fibroblasts, Cell Cycle, 16:20, 1979-1991, DOI: 10.1080/15384101.2017.1371880).
Indeed, it is slightly odd that the authors compare most of their findings with data obtained for the yeast SURF6 homologue (Rrp14), while little reference is made how/if the role of SURF6 is similar/different in humans and mice.*

We would like to thank reviewer for his time and outstanding expertise.

There are reasons why we avoided the bold comparisons between the effects of SURF6 KD/OE on ribosomal biogenesis in HeLa cells and murine fibroblasts. First, nucleolus architecture in humans and rodents are quite different, despite evolutionary closeness. Second, the effect of SURF6 KD on proliferation and survival of murine and human HeLa cells is opposite. It is likely linked to the lack of p53 which normally arrests cell cycle in response to ribosomal stress.

*Most experiments are performed to a good standard, but further analysis and/or controls could significantly improve the results and their interpretation.
Figures 1 and 2:
The inclusion of markers such as UBF, Fibrillarin or B23 (see REF 24, Yoshikawa et al.) is needed to define the sub-nucleolar localisation (FC, DFC, GC) of SURF6, which would give further insight into its role(s) in ribosome biogenesis.*

As it was mentioned in the response to the first reviewer’s comments, we performed co-staining of HeLa nucleoli with anti SURF6 and either anti-fibrillarin or anti-B23 (Fig.2 D, H, N, R). Confocal imaging shows that SURF6 pretty much co-localizes with granular nucleolar component marked by B23, while fibrillarin signal that marks FC covers smaller areas. This possibly indicates involvement of SURF6 in early, but not the earliest stages of ribosomal biogenesis.

The following text was added to the manuscript (lines 280-285 and 295-300 of revised text):

To examine potential changes in fine architecture of nucleolus, we co-stained nucleoli of control HeLa and SURF6 knockdown HeLa with anti-SURF6 and anti-fibrillarin (nucleolar fibrillar component marker) or anti-SURF6 and anti-B23 (nucleolar granular component marker) (Fig. 2C-J, M-T). Confocal images demonstrate that fine patterning of fibrillarin and B23 are not affected by SURF6 knockdown. It is interesting that fibrillarin signal is more punctate than that of SURF6 suggesting that SURF6 could potentially function outside fibrillar centers.

Further, we stained cells that overexpress SURF6-GFP fusion with ITS1 FISH probe to demonstrate that fine structure of nucleolus is not affected as well (Suppl. Fig. 2). These results suppose that overexpression of human SURF6 in HeLa does not disturb cell morphology similar to the consequence of SURF6 overexpression in mouse embryonic cells [11]. Formally, it is not possible to exclude that there are faint changes in nucleoli of HeLa cells following overexpression or knockdown of SURF6, but they would require complicated high resolution EM studies.

*While the quantitative analysis of co-localisation of SURF6 with pre-rRNA appears sound (Figure 1), it is not at all clear from the images why co-localisation of SURF6 with ITS2 is different to the other pre-rRNA segments tested?*

We agree with the reviewer that pictures of ITS2 staining look somewhat different. Quantitation and analysis using ImageJ software tool show that statistically they are very similar to results obtained with other pre-rRNA probes.

*Likewise, the phase contrast and DAPI is not sufficient to draw comprehensive conclusions about the effect of KD/OE of SURF6 on the cell (Figure 2)– for example, how does KD/OE of SURF6 affect the integrity of the nucleolus and its substructures (see above)?*

This issue was addressed by providing extra data presented in Fig. 2 (C-J, M-T) and Suppl. Fig. 2. Nucleoli of HeLa WT/KD were stained with anti-fibrillarin or B23 and SURF6 to demonstrate preserved architecture of this compartment. One can see that distribution of fibrillarin or B23 look very similar in the case of normal or downmodulated level of SURF6 in HeLa. For SURF6 OE we transfected HeLa cells with construct coding SURF6-GFP fusion and performed FISH with ITS1 probe (Suppl. Fig. 2). We failed to detect visible defects in nucleoli according to this staining.

The following text was added (lines 280-285 and 295-300 of revised text):

To examine potential changes in fine architecture of nucleolus, we co-stained nucleoli of control HeLa and SURF6 knockdown HeLa with anti-SURF6 and anti-fibrillarin (nucleolar fibrillar component marker) or anti-SURF6 and anti-B23 (nucleolar granular component marker) (Fig. 2C-J, M-T). Confocal images demonstrate that fine patterning of fibrillarin and B23 are not affected by SURF6 knockdown. It is interesting that fibrillarin signal is more punctate than that of SURF6 suggesting that SURF6 could potentially function outside fibrillar centers.

Further, we stained cells that overexpress SURF6-GFP fusion with ITS1 FISH probe to demonstrate that fine structure of nucleolus is not affected as well (Suppl. Fig. 2). These results suppose that overexpression of human SURF6 in HeLa does not disturb cell morphology similar to the consequence of SURF6 overexpression in mouse embryonic cells [11]. Formally, it is not possible to exclude that there are faint changes in nucleoli of HeLa cells following overexpression or knockdown of SURF6, but they would require complicated high resolution EM studies.

*Figure 3:
While RAMP analysis has been performed to characterise ratios between different precursors (an approach that is well-recognised in the ribosome biogenesis field), the Northern blots presented in panels A and B lack a picture of the mature rRNA levels (e.g. a methylene blue stain of the membrane) or another loading control that is not linked to ribosome biogenesis (e.g. RNase P or 7SK). Levels of the SSU processome snoRNA U3 are included – but this is not a valid loading control, given that U3 levels might change upon KD/OE of SURF6.*

We agree that U3 is not the best loading control since it’s level in some cases may be affected by changes in ribosome biogenesis. Therefore, we added pictures of ethidium bromide-stained RNA agarose gels used for Northern hybridization analysis (Fig.3 A). These images show that according to signals from 28S and 18S the amount of RNA used for Northern in SURF6 WT and SURF6 KD/OE HeLa cells was almost the same. It points that U3 snoRNA normalization we used is valid.

*How have pre-rRNA levels presented in panel D been normalised? Which statistical test has been performed to define significance?*

In panel D pre-rRNA levels were normalized to U3 snoRNA which, according to loading control, is not affected by changes in SURF6 level. Then the levels of corresponding precursors in control (scrambled siRNA-transfected) HeLa cells was considered as 1 and fold changes were plotted as histograms. Student’s t-test was used for statistical evaluation.

*While Figure 3 contains really interesting results, they need further validation before publication (as described above). In addition, both the figure itself and the description of the results are currently very confusing – as several precursors (in figure and text) and cleavage sites (in text) are mislabelled and/or used incorrectly.*

As it was mentioned before, we added another level of validation using comparison of signals from 28S/18S RNA as loading control. These data demonstrate that U3 snoRNA is not affected by SURF6 KD/OE in HeLa and can be used for normalization.

Considering mentioned mistakes in Fig.3 and text, they were corrected accordingly (see text and corrected figures in the revised version).

*For example:
Line 254/255: “Lower 21S/47S ratio also points to inhibition of site C cleavage.”
- This should be site 2, not site C.*

Changed to:

Lower 21S/47S ratio also points to inhibition of site 2 cleavage.

*Line 241/242: “It should be pointed that neither SURF6 knockdown, nor SURF6 overexpression resulted in accumulation of aberrant intermediate 34S pre-rRNA variant.”
- 34S is not present anywhere in the figure?*

This sentence was deleted from the text since the statement adds nothing to the story.

*Other specific points that should also be addressed.
Introduction:
- It would be helpful to include the pre-rRNA processing scheme in Figure 1, so that it can be referred to in the introduction.*

We would like to thank the reviewer for helpful suggestion.
We changed Fig.1 to make it easier for understanding by modifying panel D. Labels were added for all probes (ITS1/2, 18S, 28S) and their positioning was shown in bold red lines. Separate labels for ITS1 and ITS2 were added above pre-rRNA scheme.

Then, we examined the introduction and found that pre-rRNA processing and steps of this process are mentioned only twice in introduction. Removal of pre-rRNA processing scheme from Fig.3 would make it very hard for understanding. At the same time, addition of Fig.3C to Fig.1 would make it overcrowded. Using this reasoning, while feeling apologetic for arguing the reviewer, we still decided to keep initial order of figures.

*Methods:
- Please include REF numbers for the SURF6 CDS used for cloning.*

Corresponding RefSeq NM_006753.6 was added to the text.

*- Please include siRNA sequences.*

Anti-SURF6 and control siRNA duplexes (Life technologies, USA; anti-SURF6 siRNA id S13655, neg. control id am4635).

*- Please check for spelling – e.g. 1,2% agarose (instead of 1.2%) or 20MM (instead of 20 mM (Line 88).*

Thank you for finding these errors. The text was edited accordingly.

*- What is the sequence of the ITS2 probe used for Northern blotting?*

The sequence of the ITS2 probe was added to Materials and Methods, please, see section Nothern blotting.

*- Where does the ITS1 northern blot probe hybridise with respect to sites E and C?* *According the comparison of ITS1 segment and ITS1 probe sequences it needs to be clarified that ITS1 probe don’t match neither E nor C sites.*

Changes were made in Fig.3C to clarify the position of the ITS1 probe. It does not overlap with sites E or C.

*- Why is cDNA analysis described?*

We apologize for this error. We deleted the section considering cDNA synthesis since we used previously cloned cDNA to make genetic constructs mentioned in the manuscript.

*- Please provide sequences for FISH probes.*

Sequences of the probes were added to the Materials and Methods.

*Results:
- In the description of Figure 2, the text does not match the order in which the panels are described – please double-check, as KD and OE data appear to be mixed up. Which statistical test has been used to test significance in the graphs?*

Thank you for correction. We carefully checked text in the legend and corresponding figure. Please, see edited legend to Fig.2. We used Student’s t-test to confirm statistical significance.

*- Please include data (currently not shown) on cell death analysis (Line 231) or perform MTT analysis as in previous publication in mouse cells?*

Results of MTT analysis performed on HeLa (Fig.4H) and HCT116 p53+/p53- (Fig. 5D) cells with SURF6 KD and control cells. Following description was added to the main text (lines 380-383 and 397-399 in revised text):

The analysis of cell cycle itself could be misleading without evaluation of cell viability/proliferation. To close this gap, we performed MTT-test to compare viability/proliferation of SURF6 knockdown and control HeLa cells (Fig.4H). In striking contrast to murine fibroblasts, SURF6 knockdown has no negative effect on cell proliferation of HeLa cells.

According to MTT-test, proliferation/survival of HCT116 p53+ or p53- cell lines was not negatively influenced by SURF6 knockdown (Fig. 5G). Based on this data, we suggest that SURF6 affects cell cycle in HCT116 cell line largely in p53-independent manner.

*- Given that, as stated in Line 272 “These unexpected results could be explained by abnormal control and regulation of cell cycle progression in HeLa cells” – would it be useful to repeat KD of SURF6 in another human cell line, for example HCT116 cells, which, in contrast to HeLa cells, have a functional p53-signalling pathway?*

We conducted the suggested experiment in HCT116 p53+ human tumor cell line and its variant with p53 KO – HCT116 p53-. KD of SURF6 in these cell lines led to elongation of G0/G1 and shortening of G2/M (S was not affected). This finding suggests that SURF6 KD influences cell cycle independently of p53. It also supports our speculation regarding abnormal cell cycle in HeLa. Main text was chanced accordingly, please, see below. We added significant chunk of text as well (lines 388-399 of revised text):

**SURF6 knockdown affects the cell cycle in HCT116 cell line in p53-independent manner**

The question regarding a role of p53 in SURF6 effects on the cell cycle could be answered by direct comparison of cell cycle and proliferation/viability of p53 sufficient and p53 deficient cells with SURF6 knockdown. We used a pair of HCT116 cell lines which differ only in expression of the p53 protein (and designated HCT116 p53+ and HCT p53-, respectively). siRNA-mediated knockdown of SURF6 was induced in both cell lines by transient transfection with corresponding control and SURF6-specific siRNA (Fig. 5A). Then we compared the cell cycle distribution of control and SURF6 knockdown cells expressing or lacking p53. In both p53+ and p53- HCT116 SURF6 knockdown caused statistically significant increase in proportion of cells in G0/G1 phase, had no effect on S phase and markedly decreased G2/M phase (Fig. 5B-F). According to MTT-test, proliferation/survival of HCT116 p53+ or p53- cell lines was not negatively influenced by SURF6 knockdown (Fig. 5G). Based on this data, we suggest that SURF6 affects cell cycle in HCT116 cell line largely in p53-independent manner.

*Discussion:
- Line 304: “It was hypothesized that cleavages of pre-rRNA at the sites 2b and 2c of yeast ITS1 (correspond to Е and С sites in humans) are synchronized with early steps of SSU and LSU assembly (Fig 3C).”
The equivalent yeast sites are called A2 and A3, please correct.*

Corrected:

It was hypothesized that cleavages of pre-rRNA at the sites A2 and A3 of yeast ITS1 (correspond to Е and С sites in humans) are synchronized with early steps of SSU and LSU assembly

*- Line 308: “Earlie, it was shown that SURF6 could be co-immunoprecipitated with NOP52 from yeast and human cells [24].”
I could not find evidence for this in the paper.*

We thank the reviewer for the detailed analysis of presented data. This link was changed for the proper one (Horsey EW, Jakovljevic J, Miles TD, Harnpicharnchai P, Woolford JL. Role of the yeast Rrp1 protein in the dynamics of pre-ribosome maturation. RNA. 2004;10: 813–827. doi:10.1261/rna.5255804).

*- Line 318: “It has been shown earlier that disturbance of the LSU assembly may affect the* *ITS1 processing at the site 2c (corresponds to site C in humans).”
Please add a reference for this statement.*

Presented data is shown in the following paper: Wang M, Anikin L, Pestov DG. Two orthogonal cleavages separate subunit RNAs in mouse ribosome biogenesis. 2014;42: 11180–11191. doi:10.1093/nar/gku787. It’s reference #14 in the text.

- *Line 320: “We conclude that SURF6 may be required for efficient cleavage at the site C. Efficiency of the site E cleavage is not markedly affected by SURF6 knockdown.”
This is very confusing.* *The data presented in Figure 3 suggest that SURF6 is needed for site 2 cleavage, and that site E might be used in the absence of SURF6 instead.*

We agree that the interpretation that we provided is confusing. We changed the main text accordingly:

The data presented in Figure 3 suggest that SURF6 is needed for site 2 cleavage, and that site E might be used in the absence of SURF6 instead.

*Figure 3:
- Please check labelling of precursors in panels A and B (what is 17S, why is 47S not labelled here, but appears in panels D and E).*

The following reference to the article that shows detection of 17S:

Morello LG, Hesling C, Coltri PP, Castilho BA, Rimokh R, Zanchin NI. The NIP7 protein is required for accurate pre-rRNA processing in human cells. Nucleic Acids Res. 2011 Jan;39(2):648-65. doi: 10.1093/nar/gkq758. Epub 2010 Aug 26. PMID: 20798176; PMCID: PMC3025556.

Correct labeling of the 45S/45S doublet bands was added to Fig. 3A.

*- Panel C – cleavage site 4a is labelled as E on the very left.*

Corrected, please, see Fig. 3C.

*- Panel E – what is 20S? check ratios, e.g. 18S/20S – should this be 18SE/21S instead?*

Corrected, please, see Fig. 3C

*- Not all references contain the journal name, e.g. #12 and #23
- #26 and #28 are the same.*

Please, see corrected list of references.
