# Supplementary material for: Human nucleolar protein SURF6/RRP14 participates in early steps of pre-rRNA processing
Source: PLoS One. 2023 Jul 14;18(7):e0285833. doi: 10.1371/journal.pone.0285833 (PMC10348582; doi:10.1371/journal.pone.0285833)
Supplement: Attachment — Submitted filename: Response to Reviewers.docx [file pone.0285833.s006.docx]

Rebuttal letter for manuscript

PONE-D-22-03434R2

Moraleva et al. Human nucleolar protein SURF6/RRP14 participates in early steps of pre-rRNA processing

PLOS ONE

Dear
Dr. Jorge Perez-Fernandez,

First, my colleagues and I would like to thank you and both reviewers for critical evaluation of our manuscript. We are pleased to receive your kind remarks concerning the quality of data and scientific value of observations described in our study. The issues and concerns raised by the reviewers are reasonable, and it took time and effort to perform some extra experiments and analysis to answer most, if not all, points. The revised version of the manuscript is greatly improved and not only shows the involvement of SURF6 to processing of pre-rRNA, but also provides hints concerning the mechanisms that could be responsible for changes in the cell cycle caused by overexpression or downmodulation of SURF6 in human cells.

Please, find our point-by-point response to reviewers below.

Reviewers’ comments are shown in italic. Our response is in straight font. Changes in the main text of the manuscript are labeled yellow.

*Reviewer #1: In the present manuscript, the authors have presented a more comprehensive version of the functional study of Surf6. To test the function of Surf6 in Hela cells, the authors used two complementary approaches, studying the effect of depletion (via a siRNAs strategy) or overexpression of this factor on nucleolus integrity, pre-rRNA processing, and the cell cycle. With the addition of several experiments using new nucleolar markers for nucleolus staining, additional cell lines (HCT-116 p53+ and p53-) and MTT assays, the authors have fully addressed my main comments. Nevertheless, it remains quite intriguing that no proliferation defects are observed following Surf6 depletion in human Hela and HCT-116 cell lines, whereas homologs of this factor are essential in yeast and mouse.*

We thank the reviewer for this essential comment. Indeed, human SURF6 has close homologs in yeast and mice. It should be noted that despite evolutionary conservation, the organization of nucleus and smaller structures, especially nucleolus, are quite different between primitive single cell eukaryotes (yeast) and higher vertebrates such as primates and rodents (human and mouse). Thus, at least for yeast and human, we can guess that difference in effects of SURF6 knockdown or overexpression on proliferation could be explained by higher complexity of ribosomal biogenesis in humans and, possibly, development of redundant pathways, which could compensate the loss of one, or several factors involved in the pre-rRNA processing and assembly of subunits. However, this is not the case for human and mouse. Sequence homology between SURF6 and Rpr14 imply that SURF6 may have similar role in biogenesis of mammalian ribosomes. At the same time, there is a lack of information regarding molecular mechanisms that link the process of ribosomal biogenesis with the control of the cell cycle in higher eukaryotes. This makes any parallels between yeast and vertebrates speculative.

Concerning mouse and humans, we still could not rule out that difference in effects of SURF6 knockdown on cell proliferation may strongly depend on the choice of the cell line. We use immortalized or transformed cells, which usually have defects in control of the cell cycle, which could be related or independent of the p53 function. Another explanation is that rodents and primates have differences in the pathways of rRNA biogenesis.

We added substantial pieces of text to Discussion section to explain the discrepancy noted by the reviewer.

…Sequence homology between SURF6 and Rpr14 imply that human SURF6 may have similar role in biogenesis of mammalian ribosomes. Function of murine SURF6 was studied in NIH/3T3 fibroblasts [10, 11]. Zatsepina and colleagues used doxycycline inducible system to elevate Surf6 in 3T3 murine embryonic fibroblasts harboring full length Surf6 cDNA or to downmodulate Surf6 in 3T3 cells by transient transfection of genetic construct harboring antisense DNA [10, 11]. Doxycycline-induced Surf6 overexpression in 3T3 cells resulted in increased proliferation rate and altered pre-rRNA processing. Meanwhile, the antisense-mediated knockdown of Surf6 led to growth arrest and higher rate of cell death. These results demonstrated involvement of murine Surf6 in ribosome biogenesis without providing detailed molecular mechanism….

//

…Earlier, it has been reported that SURF6 knockdown in MEF 3T3 leads to elevated cell death in less than 2 days post knockdown induction [10]. In contrast to this phenotype, overexpression of SURF6 in MEF did not cause elevated cell death.

Apart from this, human nucleolar proteome is represented by at least 4500 proteins with sometimes overlapping or redundant functions (in comparison to yeast *S. cerevisiae* genome database containing only about 303 nucleolar proteins) suggesting that the ribosomal biogenesis in humans could be more flexible. This fact may help explaining the differences between S. cerevisiae which undergo cell death following RRP14 knockdown and human cells which stay proliferative following SURF6 knockdown….

*The authors discuss this observation in the last section of the “Discussion”section, and propose that this could be due to a potential role of Surf6 "in the p53-mediated growth arrest cascade". Nevertheless, the observation that mature 18S and 25S rRNA levels are not affected after Surf6 depletion argues for other hypotheses:*

*1-For some reason, Surf6 depletion is not strong enough to completely impair pre-rRNA processing, although 90% of the depletion observed by the authors should be sufficient to completely impair ribosome production.*

*2-In these cells, Surf6 function could also be supported by an additional factor with a redundant function.*

*3-In these cells, switching from pathway 1 to pathway 2 during pre-rRNA processing does not alter the efficiency of ribosome synthesis.*

*I think the authors could also insert these concepts into their discussion.*

We are grateful to the reviewer for these interesting suggestions and added required concepts to the Discussion section. It should be stressed out that our new data allow making the conclusion that the 3^rd^ scenario could be the likely explanation of the subtle effects on the ribosomal biogenesis efficiency and, hence, on the cell cycle progression. Please see the Discussion section and the text below:

…Our data suggest that in the absence of functional p53, for instance in human HeLa and HCT116 p53- cells, the shortage of SURF6 forces the usage of the pathway 1 to process pre-rRNA. Both deficit of SURF6 or its overexpression leads to accumulation of 47S/45S and 41S pre-rRNA accompanied by the decrease in 30S and 21S in HeLa cells. Similar, but less profound effects were observed in HCT116 p53- cells with SURF6 siRNA knockdown. (Fig. 4A-K). On the other side, SURF6 knockdown in HCT116 p53+ cells result in opposite effects, and cells switch the ribosome biogenesis to the pathway 2 (Fig. 3, fig. 4C, F, H, K). Our data suggest SURF6 involvement in the cleavage at the site 2 of pre-rRNA 47S/45S that initiates the pathway 2 and cleavage at site 2 in the 41S pre-rRNA in the beginning of the pathway 1. This means also that SURF6 is not required for E site cleavage in 41S and/or 21S pre-rRNAs in both pathways. Apart from this, SURF6 function may be supported with other p53-dependent factor or factors with a redundant function, which allow cells to support pathway 2 of the ribosome biogenesis without notable downregulation of the net effectiveness of this process. This hypothesis is supported by decreased level of 30S pre-rRNA along with the elevation of 18SE pre-rRNA (Fig. 4A-K) in SURF6 knockdown p53- HCT116 cells and the opposite effect on pre-rRNA processing in p53+ HCT116 [19].

We conclude that SURF6 may be required for efficient cleavage at the site 2, and in the presence of p53 its function may be supported by other factor(s) which may help cell to choose more effective ribosome biogenesis pathway to adapt to SURF6 shortage. The mechanism of pathways switching remains unknown, but the order of cleavage has been shown to vary depending on species, cell type, physiological and developmental stages. Efficiency of the site E cleavage is not markedly affected by SURF6 knockdown. This interpretation follows from ability of HeLa cells to use bypass way of 18SE pre-rRNA generation and skip intermediates 30S, 26S and 20S (see pathway 2, Fig. 3, Fig. 4). …

…Apart from this, human nucleolar proteome is represented by at least 4500 proteins with sometimes overlapping or redundant functions (in comparison to yeast *S. cerevisiae* genome database containing only about 303 nucleolar proteins) suggesting that the ribosomal biogenesis in humans could be more flexible. This fact may help explaining the differences between S. cerevisiae which undergo cell death following RRP14 knockdown and human cells which stay proliferative following SURF6 knockdown….

*Reviewer #2: The revised manuscript by Moraleva et al. is an improvement on the previous version, which addresses many major points raised, for example with respect to the subnucleolar localisation of SURF6, but some of the other points/newly included experiments require further clarification before publication.*

*Major concern:*

*The presented experiments in HeLa and HCT116 cells reveal that the effect of SURF6 KD on cell proliferation is the opposite in human and murine cells. The authors mention that this is likely linked to the lack of p53 in HeLa cells, which normally arrests the cell cycle in response to ribosomal stress. However, while this explanation makes sense for HeLa cells, it does not explain why HCT116 cells are different from murine cells (which express p53 as well), especially given the new data presented in Figure 5 saying that the effect of SURF6 KD on the cell cycle is p53 independent in HCT116 cells.*

We would like to thank Reviewer for his time and outstanding expertise. Northern blots and followed RAMP analysis were performed to evaluate the effect of the SURF6 knockdown on the pre-rRNA processing in HCT116 p53+ and HCT116 p53- cells. Following description was added to the main text (lines 322-349 of revised text).

As it was mentioned before, HeLa cells have defect in p53, meaning that it was not the best choice to study effects of SURF6 on cell cycle. Identification of the potential link between the p53-dependent cell cycle arrest caused by ribosomal stress and cell cycle could be revealed in HCT116 cell sublines which differ only in the expression of the p53 protein (and designated HCT116 p53+ and HCT p53-, respectively). siRNA-mediated knockdown of SURF6 was induced in both cell lines by transient transfection with corresponding control and SURF6-specific siRNA (Fig. 6A). The accumulation of 41S and 18SE precursors occurs in HCT116 p53- cells similar to effects we observed in HeLa cell (Fig. 4G, H). At the same time, 32S, 26S, 21S, 18SE and 12S precursors are downregulated in HCT116 p53+ cell line (Fig. 4H). This result suggests that SURF6 knockdown leads to changes in the level of different pre-rRNA precursors depending on the cell line. As mentioned above, the detailed study of changes in pre-rRNA processing requires RAMP analysis. According to RAMP, SURF6 knockdown in HCT116 p53- cell line causes changes in amounts of pre-rRNAs very similar to the that observed in HeLa cells. The most prominent are the drop in 30S/47S ratio along with the elevated 18S/21S ratio (Fig. 4K). The opposite effect was found in HCT116 p53+ cells subjected to SURF6 knockdown: 30S/47S ratio was higher, while 18SE/41S and 18SE/21S ratios were decreased in comparison to control cells (Fig. 4K). These observations allow assumption that SURF6 knockdown in p53-deficient cells switches the ribosome biogenesis from the pathway 2 to the pathway 1 (Fig. 3 and Fig.4). Opposite to this, the shortage of SURF6 in p53-positive cells facilitates the pre-rRNA processing along the pathway 2 while not causing detectable alterations in overall effectiveness of the process. Arguably, the level of p53 protein in HCT116 p53+ could be affected by SURF6 knockdown in control HCT116 p53+ cells. This possibility was ruled out by evaluation of p53 level in HCT116 p53- and p53+ cell lines following SURF6 knockdown (Fig.6A). One can see that p53 level was not affected by SURF6 knockdown.

Dependence of effects caused by SURF6 on the ribosomal biogenesis and cell cycle on p53 function could be directly proven by comparison of proliferation/viability of p53+ and p53- cells with SURF6 knockdown. We compared the cell cycle distribution of control and SURF6 knockdown cells expressing or lacking p53. In both p53+ and p53- HCT116 SURF6 knockdown caused statistically significant increase in proportion of cells in G0/G1 phase, had no effect on S phase and markedly decreased G2/M phase (Fig. 6B-F). According to MTT-test, proliferation/survival of HCT116 p53+ or p53- cell lines was not negatively influenced by SURF6 knockdown (Fig. 6G).

Based on these data, we suggest SURF6 affecting cell cycle in HCT116 cells largely in p53-independent manner. According to our guess, the presence of p53 allows cells selecting the optimal ribosome biogenesis pathway to evade cell cycle arrest under the SURF6 shortage conditions.

*As it stands, the manuscript provides pre-rRNA processing data for HeLa cells, which are not a suitable model for cell cycle analysis. On the other hand, Figure 5 shows interesting cell cycle data for HCT116 p53+ cells – but not enough information to explain how SURF6 levels impact on ribosome assembly/p53 levels/cell cycle/survival etc.*

*It would therefore be essential to check if SURF6 KD or OE in HCT116 p53+ cells indeed leads to changes in pre-rRNA processing and, as a consequence, p53 levels – which would be expected as the nucleolar stress response often seen upon ribosome assembly defects.*

Additional Northern blot, Western blot, MTT experiments as well as cell cycle analysis under SURF6 knockdown in both HCT116 p53+ and HCT116 p53- cells were performed. These data are presented in new figure 3 and discussed in the revised text:

As it was mentioned before, HeLa cells have defect in p53, meaning that it was not the best choice to study effects of SURF6 on cell cycle. Identification of the potential link between the p53-dependent cell cycle arrest caused by ribosomal stress and cell cycle could be revealed in a pair of HCT116 cell lines which differ only in the expression of the p53 protein (and designated HCT116 p53+ and HCT p53-, respectively). siRNA-mediated knockdown of SURF6 was induced in both cell lines by transient transfection with corresponding control and SURF6-specific siRNA (Fig. 6A). The accumulation of 41S and 18SE precursors occurs in HCT116 p53- cells similar to effects we observed in HeLa cell (Fig. 4G, H). At the same time, 32S, 26S, 21S, 18SE and 12S precursors are downregulated in HCT116 p53+ cell line (Fig. 4H). This result suggests that SURF6 knockdown leads to changes in the level of different pre-rRNA precursors depending on the cell line. As mentioned above, the detailed study of changes in pre-rRNA processing requires RAMP analysis. According to RAMP, SURF6 knockdown in HCT116 p53- cell line causes changes in amounts of pre-rRNAs very similar to the that observed in HeLa cells. The most prominent are the drop in 30S/47S ratio along with the elevated 18S/21S ratio (Fig. 4K). The opposite effect was found in HCT116 p53+ cells subjected to SURF6 knockdown: 30S/47S ratio was higher, while 18SE/41S and 18SE/21S ratios were decreased in comparison to control cells (Fig. 4K). These observations allow assumption that SURF6 knockdown in p53-deficient cells switches the ribosome biogenesis from the pathway 2 to the pathway 1 (Fig. 3 and Fig.4). Opposite to this, the shortage of SURF6 in p53-positive cells facilitates the pre-rRNA processing along the pathway 2 while not causing detectable alterations in overall effectiveness of the process. Arguably, the level of p53 protein in HCT116 p53+ could be affected by SURF6 knockdown in control HCT116 p53+ cells. This possibility was ruled out by evaluation of p53 level in HCT116 p53- and p53+ cell lines following SURF6 knockdown (Fig.6A). One can see that p53 level was not affected by SURF6 knockdown.

Dependence of effects caused by SURF6 on the ribosomal biogenesis and cell cycle on p53 function could be directly proven by comparison of proliferation/viability of p53+ and p53- cells with SURF6 knockdown. We compared the cell cycle distribution of control and SURF6 knockdown cells expressing or lacking p53. In both p53+ and p53- HCT116 SURF6 knockdown caused statistically significant increase in proportion of cells in G0/G1 phase, had no effect on S phase and markedly decreased G2/M phase (Fig. 6B-F). According to MTT-test, proliferation/survival of HCT116 p53+ or p53- cell lines was not negatively influenced by SURF6 knockdown (Fig. 6G).

Based on these data, we suggest SURF6 affecting cell cycle in HCT116 cells largely in p53-independent manner. According to our guess, the presence of p53 allows cells selecting the optimal ribosome biogenesis pathway to evade cell cycle arrest under the SURF6 shortage conditions.

Other points:

*• Figure 1: In agreement with reviewer 1, I also feel that Figure 1 is redundant given that much better data is provided in Figure 2.*

We agree with the Reviewer and would like to highlight that the main message of this figure and the chapter is to demonstrate co-localization of SURF6 with ITS1, ITS1, 18S and 28S regions of pre-rRNA transcripts. We added valuable data on localization of SURF6 in knockdown and overexpression experiments and presented better quality quantitative analysis of the RNA intermediates based on Nothern blot and hybridization results. Therefore, we decided to move former figure 1 to supplement section and keep the chapter in the main text to preserve the logic.

*• Figure 2: and Supplementary Figure 2: the co-localisation of SURF6 with Fibrillarin and B23 presented in Figure 2 is really informative. Why was a different, less informative method (i.e. ITS1 FISH) used to assess nucleolar structure upon SURF6 OE (Supplementary Figure 2), especially given that the figure does not show a direct comparison of control vs OE cells? It would be beneficial to analyse Fibrillarin and B23 staining upon SURF6 OE vs control cells.*

We thank the Reviewer for this comment. We set up additional ICH and confocal microscopy experiments to analyze fibrillarin, B23 and SURF6 localization in the cells with SURF6 OE. The data demonstrate normal structure of the nucleoli upon SURF6 overexpression and expected distribution and staining patterns of fibrillarin and B23 in the nucleoli. These data are presented in new figure 3 and discussed in the revised text:

SURF6 knockdown was induced by transfection of HeLa cells with siRNA specific to human SURF6 mRNA. Changes in SURF6 expression were assessed by immunoblotting of lysates obtained from SURF6 siRNA-transfected and control scramble siRNA-transfected cells according to established protocol [27]. As expected, SURF6 level dramatically decreased in SURF6 siRNA-transfected cells, but not in control cells. We observed at least 90% knockdown efficiency according to analysis of protein level (Fig. 1A, B). In contrast to mouse embryonic fibroblasts, where SURF6 siRNA knockdown caused significant changes in cell morphology and eventual cell death [10], SURF6 knockdown in HeLa had no cytotoxic effect according to MTT test neither disturbed cell morphology (Fig. 1C-R, fig. 5H). For overexpression, pcDNA3.1-hSURF6 was introduced into HeLa cells, and changes in SURF6 level in lysates from transiently transfected cells were examined 48 hours post transfection by Western blotting and immunostaining with anti-SURF6 antibodies. Lysate obtained from HeLa cells transfected in parallel with empty vector was used as negative control. As a result, the amount of SURF6 (expected single 46 kDa band) was greatly increased in lysates from HeLa/pcDNA3.1-SURF6 (Fig. 2A, B). Fluorescence intensity of nucleoli in the HeLa/pcDNA3.1-SURF6 stained with anti-SURF6 was markedly higher than that in control cells (Fig. 2C, G, K, O). According to our estimation, SURF6 level was approximately 5 times higher in HeLa/pcDNA3.1-SURF6 than in control (Fig. 2B). Microscopic images clearly demonstrate that neither overexpression nor knockdown do not affect predominantly nucleolar localization of SURF6 (Fig. 1C-R, fig. 2C-R). It should be noted that despite significant difference in SURF6 level between HeLa with overexpression and knockdown of SURF6, we failed to find detectable changes in size, shape, and pattern of DNA staining in nuclei (Fig. 1C-R, fig. 2C-R). To examine potential changes in fine architecture of nucleolus, we co-stained nucleoli of control HeLa, SURF6 knockdown and overexpression HeLa with anti-SURF6 and anti-fibrillarin (nucleolar fibrillar component marker) or anti-SURF6 and anti-B23 (nucleolar granular component marker). Confocal images demonstrate that fine patterning of fibrillarin and B23 are not affected by both SURF6 knockdown (Fig. 1C-R) and overexpression (Fig.2 C-R). It is interesting that fibrillarin signal is more punctate than that of SURF6 suggesting SURF6 could potentially function outside fibrillar centers [16]. These results suppose that overexpression of human SURF6 in HeLa does not disturb cell morphology in contrast to SURF6 overexpression in mouse embryonic cells [11]. Formally, it is not possible to exclude that there are faint changes in nucleoli of HeLa cells following overexpression or knockdown of SURF6, but their visualization would require high-resolution EM studies.

• *Figure 3: the quality of the newly included EtBr-stained gel to show levels of mature 28S and 18S rRNA is poor and not suitable to allow quantitative analysis of the U3 snoRNA, which is then used as a standard to normalise RNA levels. This experiment requires a suitable loading control for normalisation - either an RNA not involved in ribosome assembly, such as the RNA component of RNase P, as mentioned previously, or, at the very least, suitable assessment of mature 28S and 18S rRNA levels by Northern blotting.*

We agree with the reviewer that the quality of images that were used as loading and normalization controls (i.e. EtBr stained agarose gel) is poor. Therefore, we performed a new set of Northern blotting experiments and used specific oligo probe to 7SK RNA for proper loading control and normalization. New updated images of Northern hybridization experiments are presented in figure 5.

Page 2, line 59: “In contrast to primates, nucleolus in mice, despite evolutionary closeness of rodents and primates, lacks important structures.”

- This statement requires a reference.

Additional information was added to the main text (lines 61-65) of revised text with corresponding links.

In contrast to primates, nucleolus in mice, despite close evolutionary relationship of rodents and primates, lacks several intrinsic elements. It was found that there is a difference in cleavage at some pre-rRNA sites in human and mouse [12]. In primate’s nucleoli, there are species-specific proteins [13] and lncRNAs [14], which are involved in the organization of the nucleolus structure. Also, some nucleolar proteins, in particular NMP1 (protein partner of SURF6), have been found only in primates [14,15,16].

*Page 12, line 345: “utilization of E site is prevented by SURF6 depletion”.*

*- This should say: utilization of E site is promoted by SURF6 depletion”.*

Changed to (page 14, lines 404-405):

It is possible that interruption of the LSU maturation causes the disbalance and facilitates the incision at the site E.

*Page 12, line 353, 367, 368: “It was hypothesized that cleavages of pre-rRNA at the sites A2 and A3 of yeast ITS1 (correspond to Е and С sites in humans)”.*

*- Please replace “site C” with “site 2”.*

Changed to (page 14, lines 390-392):

It was hypothesized that cleavages of pre-rRNA at the sites A2 and A3 of yeast ITS1 (sites E and 2 in humans) are synchronized with early steps of SSU and LSU assembly.

*“Suppression of the SURF6 also inhibits the cleavage at the site C, but not at the site E of ITS1.”*

*- Please replace “site C” with “site 2”.*

Changed to (page 14, lines 403-404):

Suppression of the SURF6 also inhibits the cleavage at the site 2, but not at the site E of ITS1.

*“It has been shown earlier that disturbance of the LSU assembly may affect the ITS1 processing at the site 2c (corresponds to site C in humans) [14].”*

*- Please replace “site C” with “site 2”.*

Changed to (page 14, lines 405-406):

It has been shown earlier that disturbance of the LSU assembly may affect the ITS1 processing at the site 2c in yeast (corresponds to site 2 in humans).

*Legend to Figure 5, panel H: “Stars indicate statistically significant differences between control HeLa and cells with knockdown of SURF6 in their viability/proliferation.”*

*- These are HCT116, not HeLa cells.*

The order of figures was changed in the revised version of the manuscript. Figure 5 is now figure 6. Legend to figure 6 was modified accordingly.

(A) SURF6 knockdown efficiency and p53 level in HCT116 p53+ and HCT116 p53- cell lines determined by western blotting. (B-E) Quantitation of DNA content of cells from (A) stained with propidium iodide and analyzed by flow cytometry. Representative histograms show changes in proportions of cells in different stages of the cells cycle in comparison to control cells. (F) Mean percentages of cells in each phase of the cell cycle in four independent experiments. Stars indicate statistically significant differences between control cells HCT116 p53+/SURF6+ or HCT116 p53-/SURF6+, and respective cell lines with siRNA-mediated SURF6 knockdown. (G) MTT assay results obtained in HCT116 p53+/p53- control cells and respective cell lines with SURF6 knockdown and plotted as bar graphs. Mean OD ± SEM values at 570 nm were plotted on the vertical axis. Stars indicate statistically significant differences between control HCT116 and cells with knockdown of SURF6 in their viability/proliferation (p<0,05).
