# Supplementary material for: Human nucleolar protein SURF6/RRP14 participates in early steps of pre-rRNA processing
Source: PLoS One. 2023 Jul 14;18(7):e0285833. doi: 10.1371/journal.pone.0285833 (PMC10348582; doi:10.1371/journal.pone.0285833)
Supplement: Attachment — Submitted filename: Response to Reviewers PONE-D-22-03434R3.docx [file pone.0285833.s007.docx]

Rebuttal letter for manuscript

PONE-D-22-03434R3

Moraleva et al. Human nucleolar protein SURF6/RRP14 participates in early steps of pre-rRNA processing

PLOS ONE

Dear
Dr. Jorge Perez-Fernandez,

First, my colleagues and I would like to thank you and both reviewers for critical evaluation of our manuscript. We are pleased to receive these kind remarks concerning the quality of data and scientific value of our study. Concerns raised by the reviewers are reasonable, and some additional experiments and analysis were performed. The revised version of the manuscript contains statistical analysis of the p53 level in HCT116/p53+ cell line comparing cells transfected with scrambled control RNA and siRNA targeting transcript of TP53 gene. Result of this experiment demonstrates no significant difference between control cells and cells with siRNA-induced p53 knockdown. Point-by-point response to minor points is included as well. Please, find our point-by-point response to reviewers below.

Reviewers’ comments are shown in italic. Our responses are in straight font. Changes in the main text of the manuscript are labeled yellow.

*Reviewer #1: In this last version of the manuscript, authors have corrected their manuscript in attempt to answer to our comments. For example, discussion section has been modified to clarify the discrepancies observed after SURF6/Rrp14 depletion in yeast, mice and human cells. Nevertheless, as it stands, it is still rather difficult to interpret some piece of data. Indeed, in both p53+ and p53- HCT_116 cells, SURF6 knock-down promote G1/G0 arrest and a G2/M reduction but do not have any effect on cell death (see MTT assays). Rather, it seems MTT signal is greater after 3 days, indicating a greater proliferation after SURF6 knock-Down in these cells. Although, this work is a good attempt to understand how Surf6 affects ribosome production and/or cell cycle in humans cells. More work will be required to fully decipher the function of this factor.*

We thank the reviewer for critical comments. In regards to G1/G0 arrest, it is worth noting that observed minor changes in cell numbers likely not sufficient to cause substantial effects on proliferation and/or cell death rates. Apart from this, little is known about possible functions of SURF6 that are not related to ribosome biogenesis. Our data and previous reports show slight increase in proliferation rate due to knockdown of SURF6. These observations are counter intuitive since one would expect the opposite. Nonetheless, we cannot exclude potential secondary/indirect effects caused by changes in SURF6 level on cell proliferation and not linked to ribosome biogenesis. We absolutely agree that our data clearly demonstrate the influence of SURF6 on ribosome biogenesis, but dissection of molecular mechanism underlaying the links between SURF6, p53, ribosome biogenesis and cell cycle regulation goes far beyond the scope of our study. It would require separate study in a near future.

*Reviewer #2: The additional experiments included in the revised manuscript by Moraleva et al. (i.e. the comparison of HCT116 p53+ and p53- cell lines, the use of 7SK as a loading control for Northern Blot quantification and the use of specific marker proteins to assess the sub-nucleolar structure upon SURF6 knockdown/overexpression) have fully addressed my previous concerns. In agreement with reviewer 1, I also find it quite intriguing that no proliferation defects are observed following SURF6 depletion in human Hela and HCT116 cell lines, as opposed to the situation in yeast and mouse. However, sufficient detail is now provided in the discussion to highlight this discrepancy.*

We thank the reviewer for the careful evaluation of our manuscript. *Minor points:

Line 118
….sample (pre-rRNA 34S/36S, 12S/32S etc) …
- According to Figure 3, there is no 34S – please correct.*

Changed to pre-rRNA 41S/45-47S, 12S/32S etc.  *Line 119
… log2 values of precursors ratios in the control samples (untreated HeLa cells) …
- This should now say (untreated HeLa and HCT116 cells).*

Changed to untreated HeLa and HCT116 cells. *Line 128
…. determined by Bredford reaction…
- This should say Bradford.*

Changed to Bradford. *Line 337
… One can see that p53 level was not affected by SURF6 knockdown.
- This is quite a surprising and important result. The statement should therefore be supported by quantification and statistical analysis of p53 levels in several repeat knockdown experiments.*

We thank the reviewer for reasonable concern regarding reproducibility of results. Additional experiments were conducted to solve the issue (resulting images of western blot and immunostaining are presented in the raw data file). Statistical analysis was based on three independent siRNA transfection experiments and is shown in figure 6 B. According to the results, there are no statistically significant differences in the p53 level between control cells and cells with SURF6 knockdown. Corresponding changes were made in the legend to figure 6 and experimental section of the manuscript

*Line 356
…Sequence homology between SURF6 and Rpr14…
- Rrp14*

Changed to Rrp14. *Line 368
…Sequence homology between human SURF6, murine Surf6 and yeast Rpr14 ...
- Rrp14*

Changed to Rrp14. *Lines 402/403
… It has been shown earlier that disturbance of the LSU assembly may affect the ITS1 processing at the site 2c in yeast (corresponds to site 2 in humans) [19].
- Should this say “processing at the site A3 in yeast (corresponds to site 2 in humans)” ?*

Changed to: “It has been shown earlier that disturbance of the LSU assembly may affect the ITS1 processing at the site A3 in yeast (corresponds to site 2 in humans)”.
